# A systematic analysis of marine lysogens and proviruses

**DOI:** 10.1038/s41467-023-41699-4

**Published:** 2023-09-27

**Authors:** Yi Yi, Shunzhang Liu, Yali Hao, Qingyang Sun, Xinjuan Lei, Yecheng Wang, Jiahua Wang, Mujie Zhang, Shan Tang, Qingxue Tang, Yue Zhang, Xipeng Liu, Yinzhao Wang, Xiang Xiao, Huahua Jian

**Affiliations:** 1 State Key Laboratory of Microbial Metabolism, Joint International Research Laboratory of Metabolic & Development Sciences, School of Life Sciences and Biotechnology, Shanghai Jiao Tong University, Shanghai, China; 2 Yazhou Bay Institute of Deepsea Sci-Tech, Shanghai Jiao Tong University, Sanya, China; 3 Southern Marine Science and Engineering Guangdong Laboratory (Zhuhai), Zhuhai, China

**Keywords:** Bacteriophages, Microbial ecology, Phage biology, Virus-host interactions, Water microbiology

## Abstract

Viruses are ubiquitous in the oceans, exhibiting high abundance and diversity. Here, we systematically analyze existing genomic sequences of marine prokaryotes to compile a Marine Prokaryotic Genome Dataset (MPGD, consisting of over 12,000 bacterial and archaeal genomes) and a Marine Temperate Viral Genome Dataset (MTVGD). At least 40% of the MPGD genomes contain one or more proviral sequences, indicating that they are lysogens. The MTVGD includes over 12,900 viral contigs or putative proviruses, clustered into 10,897 viral genera. We show that lysogens and proviruses are abundant in marine ecosystems, particularly in the deep sea, and marine lysogens differ from non-lysogens in multiple genomic features and growth properties. We reveal several virus-host interaction networks of potential ecological relevance, and identify proviruses that appear to be able to infect (or to be transferred between) different bacterial classes and phyla. Auxiliary metabolic genes in the MTVGD are enriched in functions related to carbohydrate metabolism. Finally, we experimentally demonstrate the impact of a prophage on the transcriptome of a representative marine *Shewanella* bacterium. Our work contributes to a better understanding of the ecology of marine prokaryotes and their viruses.

## Abstract (web summary)

Viruses are ubiquitous in the oceans, exhibiting high abundance and diversity. Here, Yi et al. present a systematic catalogue and analysis of genomic sequences from marine prokaryotes and their proviruses, thus contributing to a better understanding of the ecology of these microorganisms.

## Introduction

Viruses are ubiquitous in natural environments, and their extremely high abundance, diversity, and activity make them indispensable to various Earth’s ecosystems^1^. Viruses can be generally classified as virulent or temperate; the latter are those viruses that are capable of both lytic and lysogenic infections. During its lysogenic cycle, a temperate virus usually integrates into the host genome to form a provirus or prophage, the latter of which refers to a provirus integrated into the bacterial genomes^2,3^. Prophages are widespread in bacterial genomes^4^, and the prevalence of temperate virus-infected microbes, designated lysogens, varies in different environments^5,6^. Notably, multiple lines of evidence have suggested that temperate viruses and lysogens are prevalent in the ocean, which is the largest ecosystem on Earth^7–9^. Recently, Tuttle et al. analysed 1239 marine bacterial genomes and found that 17.7% of these genomes contained at least one prophage^8^. Furthermore, multiple viral genome-harbouring integrases have been identified from Mediterranean seawater, indicating that temperate viruses may be widely distributed in the deep sea^10^. Correspondingly, several temperate viruses have been isolated and members of which can infect dominant marine microbes, such as SAR11 and cyanobacteria^11,12^. Despite these important advances, most studies either had relatively small sample sizes or focused on culturable microorganisms, thus likely underestimating the occurrence of lysogeny in the oceans^8^.

Temperate viruses significantly influence a variety of physiological functions in host microorganisms, including DNA replication, gene transcription, protein expression, growth, motility, biofilm formation, and resistance to environmental stressors^2,3,13,14^. Importantly, the cryptic prophages, although they cannot produce intact phage particles, still confer significant benefits to the host^15,16^. Despite being a “time bomb” that can cause host cell death, temperate viruses are also pivotal for the survival of marine bacteria^7,17–20^. Led by the breakthrough finding that cyanophages possess photosynthesis genes^21^, researchers have identified and characterized a variety of auxiliary metabolic genes (AMGs) encoded by marine viruses^22,23^. Specifically, multiple AMGs were found in the Pacific Ocean Virome (POV) and Global Oceans Virome (GOV)^24,25^, and viral-encoded reverse dissimilatory sulfite reductase was discovered in hydrothermal vent plumes from the Lau and Guaymas basins, suggesting that viruses participate in oceanic sulfur cycling^26^. To date, although the genomic content and context of AMGs have been investigated in few marine viral clades, such as roseophages^27^, the occurrence and functional category of marine temperate virus-encoded AMGs warrant systematic assessment.

In general, although an increasing number of studies have suggested that lysogeny is widely distributed in the ocean and that temperate viruses may mediate various biogeochemical processes^7,8,22^, a comprehensive description and quantitative assessment of the genomic features and ecological functions of marine temperate viruses is currently lacking. Here, based on a large-scale analysis of marine prokaryotic genomes and their accompanying environmental parameters, we constructed a Marine Temperate Viral Genome dataset (MTVGD). Subsequently, we systematically assessed the distribution, diversity and potential ecological functions of marine temperate viruses in oceans worldwide.

## Results and discussion

### The distribution patterns of lysogens in global oceans

**Fig. 1:**
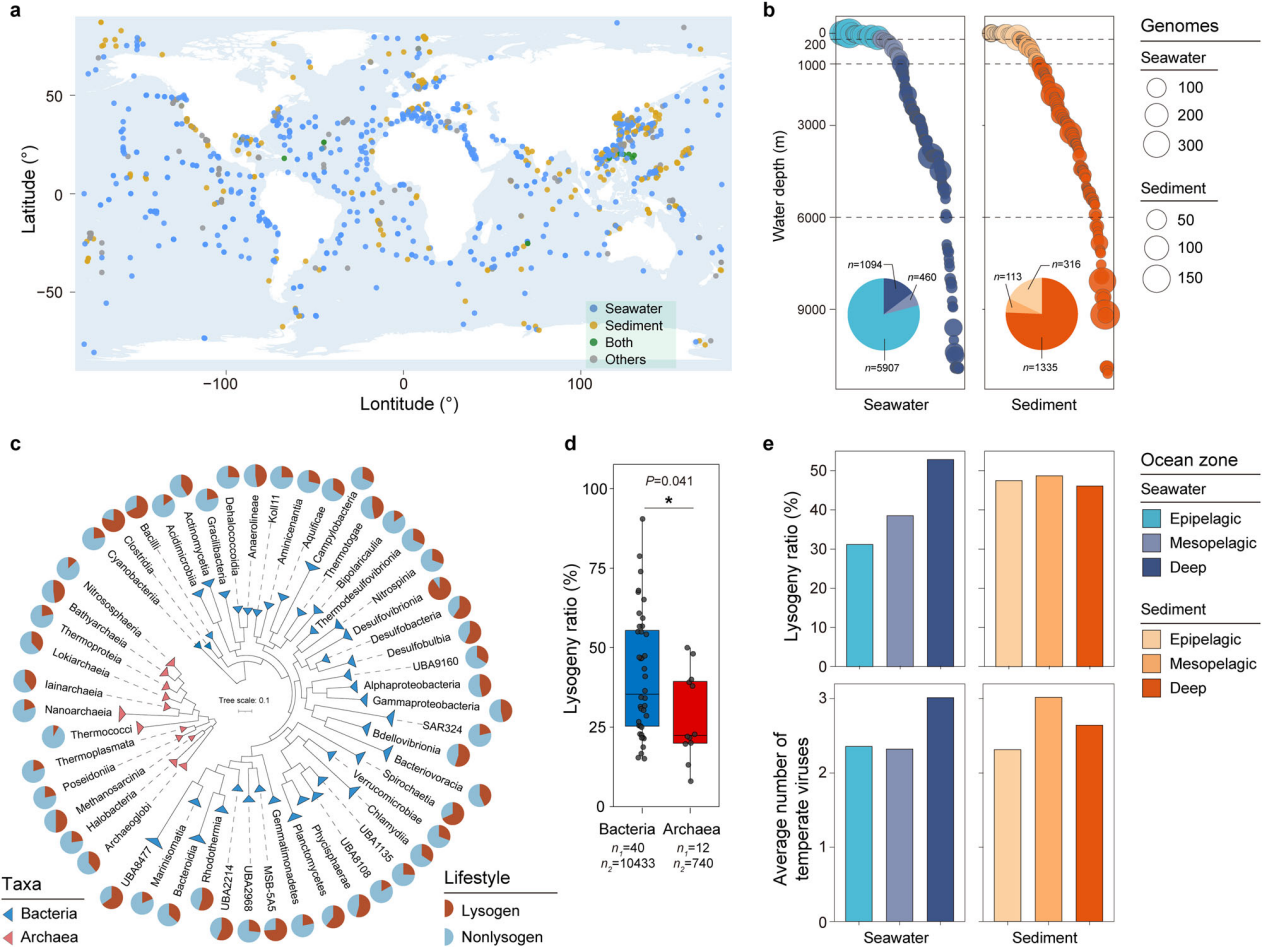
Overview of the Marine Prokaryotic Genome dataset (MPGD) and the lysogeny landscape in the ocean. **a** Geographic distribution of genomes in the MPGD. Each point represents a geographic site, and the colour indicates the genome derived environment: seawater, sediment, both or others (information unavailable). The map was drawn using the R package maps (3.4.1)^126^, in which the “world” data derived from the Natural Earth (v2.0) (https://www.naturalearthdata.com/). **b** Water depth distribution of genomes in the MPGD. Each circle in the scatter plots shows the water depth, and the circle size is proportional to the number of genomes found at that depth. The pie chart shows the proportions of genomes sampled from different ocean zones: the epipelagic (water depth: 0–200 m), mesopelagic (200–1000 m) and deep-sea (>1000 m) zones. **c** Ratios of lysogeny in different marine prokaryotic taxa. The bacterial and archaeal phylogenetic trees were constructed based on 120 and 122 concatenated marker proteins, respectively, using the maximum-likelihood algorithm. All branches were collapsed at the class level. Each pie chart corresponds to a class (with ≥ 20 genomes), showing the proportions of lysogenic and nonlysogenic genomes. **d** Comparison of lysogeny ratios between bacteria and archaea at the class level. *n*_*1*_: number of prokaryotic classes; *n*_*2*_: number of prokaryotic genomes contained in these classes. The significant difference between LyRs of bacterial and archaeal classes was determined by a two-sided Wilcoxon rank-sum test, and the *P*-value is shown above boxes. Each box represents the interquartile range (IQR), in which the middle line represents the median. The whiskers extend to 1.5 × IQR, and all contained data are shown as the individual points. **e** Occurrence of lysogeny in different ocean zones. Source data are provided as a Source Data file.

To construct the Marine Prokaryotic Genome dataset (MPGD), we extracted data from the NCBI GenBank database^28^, the Genomes from Earth’s Microbiomes (GEM)^29^ and Oceanic Trench Microbial Genomes (OTMG)^30^ datasets. The MPGD comprises 11,148 bacterial and 932 archaeal genomes with high quality (completeness > 80%, contamination < 5%); it covers 215 classes and 1793 genera (Supplementary Fig. 1, 2 and Data 1). Notably, the MPGD covers various ocean regions, including the Pacific, Atlantic and Indian Oceans (Fig. 1a), and the full ocean depth (from 0 to 11,094 m) (Fig. 1b). Among the genomes in the MPGD, 71.7% and 16.7% were derived from seawater and sediment, respectively. Of the seawater-derived genomes, the majority (79.2%) originated from the epipelagic zone, followed by the deep sea (14.7%) and mesopelagic zone (6.2%) (Fig. 1b). Of the marine sediment-derived genomes, the majority (75.7%) were from the deep sea. In total, we recovered 3351 genomes from the aphotic zone (water depth ≥ 1000 m), including 381 (11.4%) originating from the hadopelagic zone (water depth ≥ 6000 m), thus providing an important dataset to evaluate lysogens in the dark ocean.

Subsequently, we identified and curated temperate viral sequences in the MPGD (Supplementary Data 2 and 3) to construct the MTVGD, which comprises 12,918 viral contigs (Supplementary Data 4). Notably, 1915 contigs could be recruited by the marine viromes (Supplementary Fig. 3), thereby providing evidence for their dynamic activity in the ocean. Containing 205,027 protein clusters (PCs), the viral contigs were clustered into 10,936 viral operational taxonomic units (vOTUs), showing high diversity of marine temperate viruses. Compared with proviruses in the current largest viral genome database IMG/VR v3^31^, we found that 96.4% of vOTUs and 83.9% of PCs in the MTVGD were novel, thus identifying 16.7% and 11.5% more vOTUs and PCs, respectively, to contribute to the proviral genomes worldwide. Despite this progress, the viral PCs in the MTVGD were far from saturated (Supplementary Fig. 4), suggesting that unprecedented diversity of marine temperate viruses warrant further exploration in the future.

To place viruses with multiprotein-based related phylogeny into clusters, a total of 7964 viral sequences ≥ 10 kb were grouped by vConTACT2 (Supplementary Data 5); 4727 of them were allocated into 1,151 temperate viral clusters (tVCs) (Supplementary Data 6), in which only 8.7% were clustered with reference sequences. Notably, 5 tVCs contained viruses with genome sizes > 200 kb (Supplementary Fig. 5); these viruses probably belong to the huge phage clades^32^. Regarding taxonomic composition, only 13.6% and 4.2% of total proviral genomes could be identified at the class and family levels, respectively. *Peduoviridae* (43.1%), *Kyanoviridae* (24.3%) and *Autographiviridae* (16.4%) made up the majority of viral family assigned genomes (Supplementary Fig. 6).

In total, 4880 genomes in the MPGD contained at least one viral sequence (mean = 2.65), indicating a high lysogeny ratio (LyR, 40.4%) among marine microbes. This overall LyR was significantly higher than that in a previous report, and was consistent with their speculation that they may underestimate the true occurrence of lysogeny^8^. We acknowledge that the LyR could still be underestimated due to the majority of partial genomes in the MPGD, as it reached 46.5% and 56.1%, when calculated in genomes with 90% ~ 100% and 100% completeness, respectively (Supplementary Fig. 7).

Different taxa of marine microorganisms showed divergent LyRs (Fig. 1c, Supplementary Data 7), ranging from 8.0% (Thermococci) to 90.5% (Desulfovibrionia) at the class level. Bacteria showed significantly higher LyRs (mean = 41.2%) than archaea (mean = 28.5%) at the class level (*P* = 0.0412) (Fig. 1d). In terms of bacteria, Alpha and Gammaproteobacteria, the dominant microbes in the MPGD, showed LyRs of 46.7% and 46.6%, respectively. Compared to most classes, Desulfovibrionia, Desulfobacteria, Desulfobulbia and Bacilli displayed higher LyRs, while Cyanobacteriia and Bacteroidia displayed lower LyRs. As for archaea, Methanosarcinia and Bathyarchaeia exhibited relatively high LyRs, whereas Nitrososphaeria and Poseidoniia exhibited lower LyRs (Fig. 1c).

Overall, the LyRs of prokaryotes in marine sediments (48.1%) were higher than those in seawater (38.4%). Interestingly, the LyRs of seawater-derived prokaryotes increased with water depth, reaching 52.8% in deep seawater, while they remained nearly constant in sediment-derived microbes, ranging from 46.1% to 48.7% (Fig. 1e). These trends were consistent in different ranges of genome completeness (Supplementary Fig. 7). We further found that microbes derived from deep-sea water and sediments exhibited significantly higher LyRs (Supplementary Fig. 8 and Data 8). Moreover, the average number of temperate viral sequences carried per lysogenic genome was higher in deep-sea water (mean = 3.01) and sediment (mean = 2.64) than in epipelagic seawater (mean = 2.36) and sediment (mean = 2.31) (Fig. 1e). In accordance with previous studies^30,33,34^, these results indicate that lysogeny is probably more prevalent in the deep sea.

Among the marine lysogens, 35.4% were polylysogens that contained multiple proviruses (Supplementary Fig. 9). Intriguingly, two polylysogens, *Hyphomonas* sp. IN11 and *Dehalococcoidia* sp. UBA6537 harboured 18 and 23 proviruses in their genomes, respectively (Supplementary Fig. 10). The incidence of polylysogeny among different marine environments showed a similar pattern to that of lysogeny (Fig. 1e and Supplementary Fig. 9), while lysogens in mesopelagic sediment showed the highest polylysogeny ratio (43.2%). Although the occurrence of polylysogeny was relatively low in deep-sea sediment (30.1%), polylysogens in that habitat seemed to harbour more temperate viruses (mean = 3.01) (Supplementary Fig. 9). Synthesizing these results, we propose that the lysogenic lifestyle is prevalent in global oceans, especially in the deep sea.

### Genomic features and life-history traits of marine lysogens and temperate viruses

**Fig. 2:**
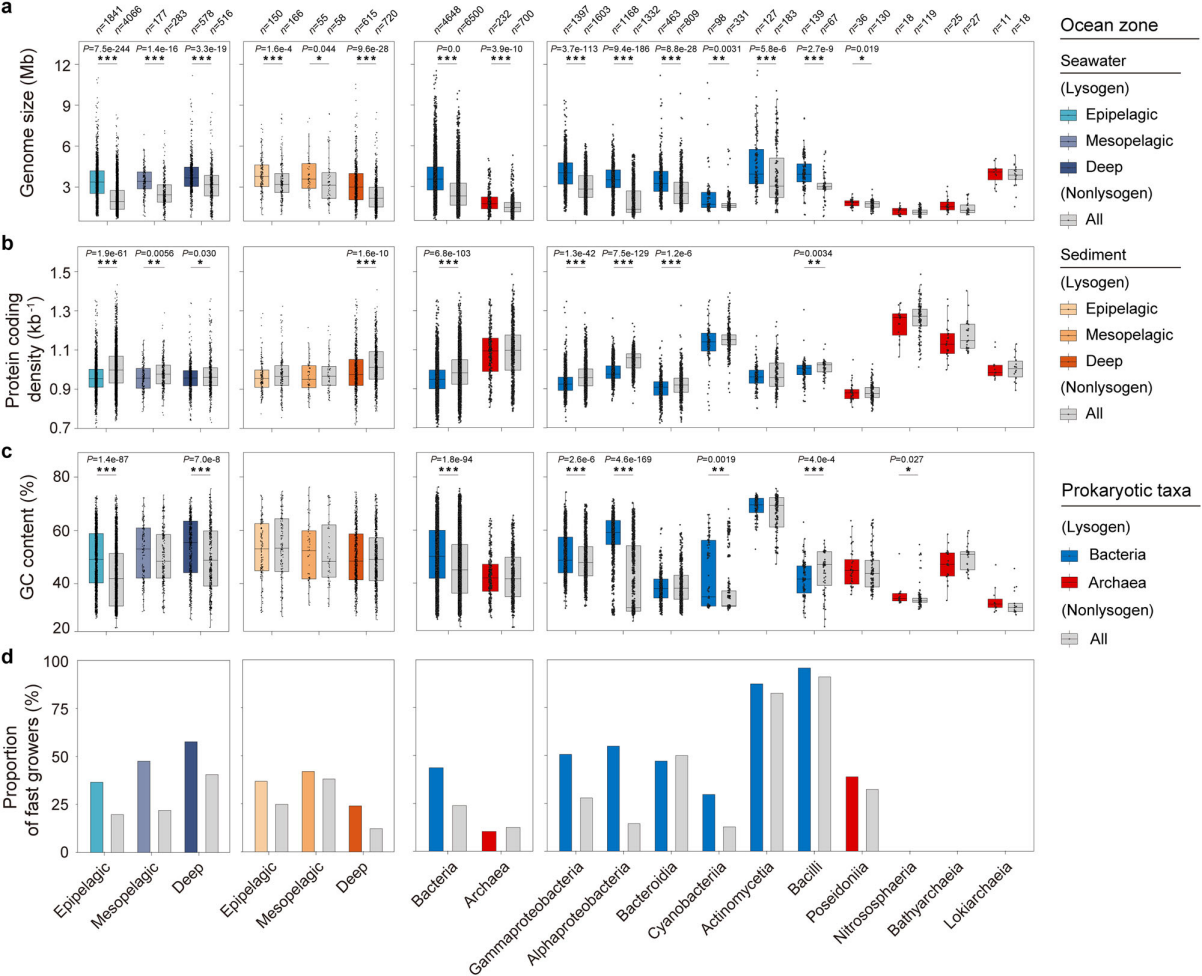
Comparison of genomic features and growth traits between marine lysogens and nonlysogens. **a**–**c** Box plots representing the genome size (**a**), protein coding density (**b**), and GC content (**c**), of marine lysogens and nonlysogens. All parameters of lysogens were calculated based on genomes with temperate viral sequences excluded. All the significant differences between lysogens and nonlysogens were determined by two-sided Wilcoxon rank-sum tests, and the *P-*values are shown above boxes. Each box represents IQR, in which the middle line represents the median. The whiskers extend to 1.5 × IQR, and all contained data are shown as the individual points. **d** Coloured and grey bars represent the percentage of fast growers (MDT < 5 h) in marine lysogens and nonlysogens, respectively. The marine prokaryotes were grouped based on ocean zone (left two panels) or host taxon (right two panels). Data groups in the (**a**–**d**) shared the same sample sizes (number of genomes in each group) which were shown at the top of the (**a**). Source data are provided as a Source Data file.

Given these findings, we then analysed the divergence of genomic features between the lysogenic and nonlysogenic groups (Supplementary Data 9). Marine lysogens often displayed significantly larger genome sizes, higher guanine-cytosine (GC) content and nitrogen atoms per residue side chain (N-ARSC), as well as lower protein coding density (PCD) and fewer carbon atoms per residue side chain (C-ARSC) than nonlysogens (Fig. 2a–c and Supplementary Fig. 11), while as exceptions, N-ARSC and C-ARSC showed opposite differences in archaea and several bacterial classes, such as Bacteroidia and Bacilli (Supplementary Fig. 11). Interestingly, divergences seemed to occur more frequently and markedly in seawater than sediment, and in bacteria than archaea. The largest differences in genome size and PCD between lysogens and nonlysogens occurred in surface seawater and deep-sea sediment (Supplementary Fig. 12).

To determine whether marine lysogens have life-history traits that have differentiated from those of nonlysogens, we estimated the minimum doubling time (MDT) of marine prokaryotes, and calculated the percentage of fast growers (MDT < 5 h) in different groups (Fig. 2d and Supplementary Data 10). We found that lysogens generally had a higher percentage of fast-growers than nonlysogens. The exceptions were archaea and Bacteroidia, in which the percentage of fast-growers in lysogens was slightly lower than that in nonlysogens. Furthermore, among the fast growers, the MDT of bacterial lysogens was significantly lower (*P* = 3.7e-10) than that of nonlysogens; the opposite pattern was observed in marine archaea (Supplementary Fig. 13).

To further explore the extent to which genomic features were associated with the prevalence of marine lysogens, we performed a correlation analysis between the genomic features and LyRs (at the genus level) (Supplementary Data 11). We found that genome size, GC content and N-ARSC generally showed a positive correlation with LyR, while PCD and C-ARSC generally displayed a negative correlation with LyR (Supplementary Fig. 14). Notably, genome size was outstandingly significantly correlated with LyR across different marine environments and for almost all abundant prokaryotic taxa. These shared correlations suggest that marine lysogens have unique genomic features, and thus are physiologically and metabolically distinct from nonlysogens. Although this speculation has been confirmed in a few pure cultures of marine bacteria^17,19,20,35^, further high-throughput and multispecies data are needed.

In addition to marine lysogens, we further exploited the genomic differentiation of marine temperate viruses (Supplementary Data 12). The water depth-dependent evolution and differentiation of viral populations has previously been observed in the Mediterranean Sea, North Pacific Subtropical Gyre and hadal trenches^10,30,36^. Here we showed, interestingly, that the GC content of seawater-derived temperate viruses first increased and then decreased with increasing water depth, while the C-ARSC showed an opposite trend (Supplementary Fig. 15). This phenomenon is in accordance with a previous report of a microbial genomic transition zone below the photic zone^36^, suggesting that temperate virus-host coevolution influenced their genomic features. For sediment-derived temperate viruses, differences in genomic features were also associated with water depth but showed diverse trends (Supplementary Fig. 15). However, temperate viruses from different depth-stratified zones did not significantly differ in N-ARSC, suggesting that nitrogen source did not influence this divergence to the same extent.

### Interaction networks of marine temperate viruses and prokaryotes

**Fig. 3:**
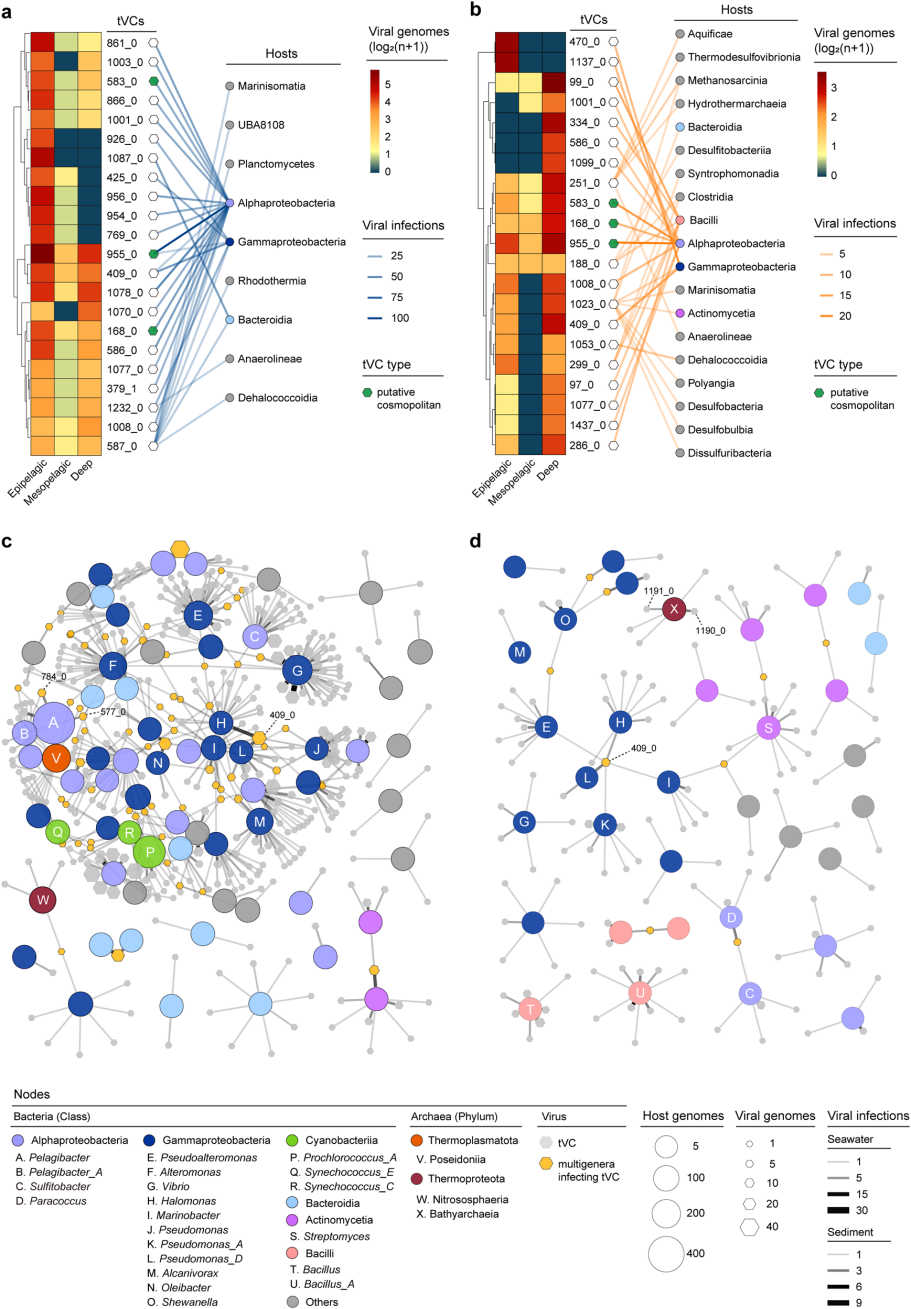
Interactions between marine temperate viruses and prokaryotic hosts. **a**, **b** Distribution and host ranges of temperate viral clusters (tVCs) in seawater (**a**) and sediment (**b**). For clarity, only tVCs with ≥15 (in seawater) or ≥5 (in sediment) viral genomes are shown in the heatmaps. The heatmaps show the number of viral genomes in tVCs derived from 3 depth-stratified ocean zones, which are hierarchically clustered by tVCs. The tVCs present in all 6 ocean zones are considered as putative cosmopolitan tVCs and are marked in green next to the tVC names. Each tVC is connected to the class of its host(s), and the transparency of the connecting lines is proportional to the number of infections. **c**, **d** Interaction networks of prokaryotic genera and tVCs in seawater (**c**) and sediment (**d**). For clarity, only prokaryotic genera with ≥20 genomes (in seawater) or ≥5 genomes (in sediment) are displayed in the network. The circles and hexagons represent host genera and tVCs, respectively, and the sizes are proportional to the numbers of genomes included. The coloured circles represent different bacterial classes or archaeal phyla, the inner letters mark ecologically relevant marine microbial hosts (at the genus level), and the yellow hexagons represent tVCs that infect multiple host genera. The numbers of infections are displayed as the shared edges and proportional to the transparency and width. The networks are visualized using the edge-weighted spring-embedded model, which places the host genera and tVCs sharing higher co-occurrence in closer proximity. Source data are provided as a Source Data file.

To identify ecologically important marine temperate viruses, we initially focused on enriched tVCs that encompassed ≥15 and ≥5 viral genomes in the seawater and sediment groups, respectively. Then, the distribution and host ranges of these tVCs were analysed; they were shown to be distinct in sediment and seawater and to differ in distribution in different ocean zones (Fig. 3a, b). Several tVCs (tVC 583_0, 168_0, and 955_0) were ubiquitous in all groups, suggesting that they may be cosmopolitan tVCs in the ocean. Then, to illustrate the marine temperate virus-host interactions, we included the enriched prokaryotic genera (with ≥20 and ≥5 genomes in the seawater and sediment groups, respectively) and resident tVCs in interaction networks (the complete networks are provided in Supplementary Figs. 16 and 17). Notably, the networks showed significantly more frequent virus-host interactions in seawater than in sediment (Supplementary Data 13). Subsequently, we identified the tVCs infecting ecologically important marine prokaryotic clades (Fig. 3c, d). Among the seven *Pelagibacter*-infecting tVCs, two unclassified tVCs, 784_0 and 577_0, showed frequent infections, which was represented by a high frequency of these temperate viruses present in the genomes of a host genus. tVC 784_0 contains the recently reported prophage PNP1^11^, and shares homologous proteins with the highly abundant SAR11 virus HTVC010P; tVC 577_0 clustered with several previously reported SAR11 viruses (HTVC011P and HTVC019P)^37^ (Supplementary Fig. 18). Notably, in addition to SAR11, tVC 577_0 can infect multiple genera of Gammaproteobacteria. For archaeal viruses, two novel tVCs, 1190_0 and 1191_0, were found to frequently infect Bathyarchaeia (Fig. 3d), which is the critical driver of the carbon cycle in marine sediment^38^. Both of them carried head and tail proteins homologous to bacteriophages, and TerL homologous to archaeal virus HGTV-1 (Supplementary Fig. 19).

Although most tVCs show narrow host ranges (Supplementary Fig. 20), we identified 301 and 68 multigenera-infecting tVCs in seawater and sediment, respectively (Fig. 3c, d, Supplementary Figs. 16 and 17). Notably, tVC 409_0, which was classified in the family *Peduoviridae*, was highly enriched (Fig. 3a, b), infected 15 and 9 genera in seawater and sediment, respectively, and interacted with multiple Gammaproteobacteria hosts (Fig. 3c, d, Supplementary Fig. 21), which have been reported to prevail in diverse marine environments^39,40^. To conservatively evaluate broad-host-range viruses, we classified MTVGD into 10,897 temperate viral genera (tVG) using VIRIDIC^41^, and found 30 multiphylum-infecting and 37 multiclass-infecting tVG, represented by 5 tVG infecting Gammaproteobacteria and other classes (Supplementary Fig. 22). Some of the cross-phylum/class infectivity were further supported by host prediction based on virus-host homology of CRISPR spacer or nucleotide sequence (Supplementary Fig. 22). Host DNA can be carried by temperate viruses and transferred to other hosts via the process of transduction, which is an important mechanism of provirus-mediated horizontal gene transfer (HGT)^42^. Among diverse variables, the host range is a key variable that affects the ability of virus-mediated HGT^43^. Thus, we propose that tVCs/tVG with broad host ranges could potentially be important mediators of HGT in marine ecosystems. Finally, by analysing the co-occurrence of different tVCs in the same host, we observed significantly intertwined coinfections among tVCs, especially in seawater (Supplementary Fig. 23 and Data 14), indicating their high compatibility and potential mutual effects.

Leveraging the viral-host interaction analysis, we revealed putative ecologically important tVCs/tVG, including those that (1) infect dominant marine microbial clades (such as SAR11, Gammaproteobacteria and Bathyarchaeia); (2) are enriched in multiple environments and thus potentially cosmopolitan in the ocean; and (3) feature extraordinarily wide host ranges and are speculated to be important in mediating genetic exchanges. Previously, multiphylum-infecting phages were isolated from Lake Michigan, and these phages are capable of infecting 5 strains belonging to 3 different bacterial phyla including Proteobacteria, Actinobacteria and Bacteroidetes^44^. Moreover, metagenome-based studies and CRISPR matches have found viral clusters linked to multiple hosts from different phyla^45^. Especially, in marine environments, broad-host-range viruses are reported to be distributed widely^46,47^. Recently, microbial domain-crossing host-viral interactions were illustrated in dense hydrothermal mats in the Guaymas Basin^48^. These findings suggested that despite currently no marine temperate viruses capable of infecting multiple phyla have been isolated, they are very likely to exist in the ocean. Although the vast genetic diversity of marine viruses has been documented in several fundamental datasets^31,45,49^, we believe that the aforementioned tVCs and their hosts warrant isolation and characterization, thereby revealing details of their life strategy and ecological functions.

### Ecological influences of marine temperate viruses

**Fig. 4:**
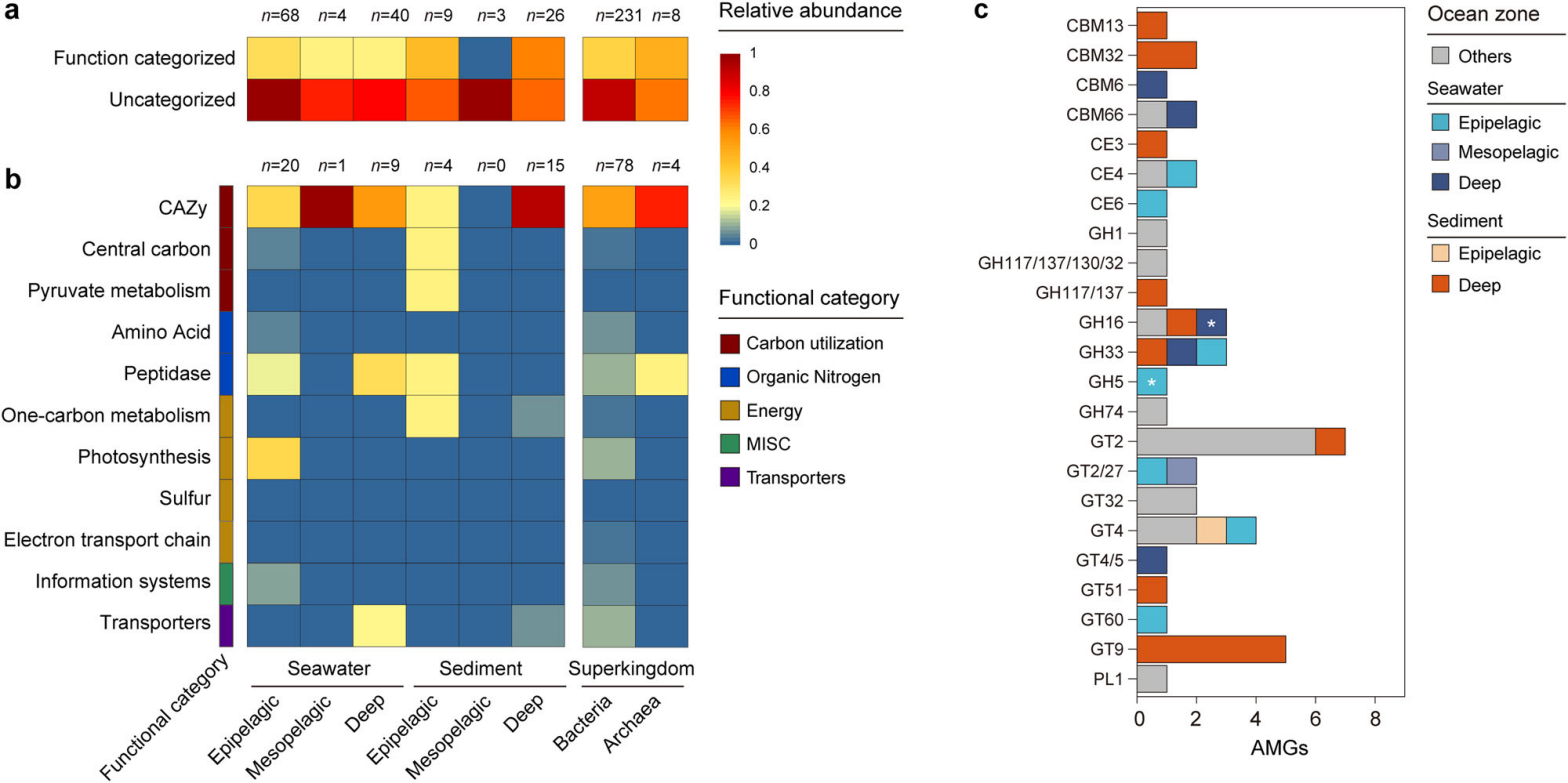
Distribution of auxiliary metabolic genes (AMGs) in marine temperate viruses. **a**, **b** Heatmap showing the relative abundance of all AMGs (**a**) and the functionally categorized AMGs (**b**) encoded by marine temperate viruses in each group. The relative abundance was calculated as the average number of AMGs carried by per viral genome. The groupings are based on the ocean zone or host taxon. Functional categories of AMGs are annotated by DRAM-v^82^. The number of viral genomes contained in each group is shown at the top of the heatmap. CAZy Carbohydrate-Active enZYmes, MISC miscellaneous. **c** Composition of AMGs involved in CAZy. The asterisks indicate that AMGs were functionally characterized in this study. CBM *c*arbohydrate-binding module, CE carbohydrate esterase, GH glycoside hydrolase, GT glycosyl transferase, PL polysacchaide lyase. Source data are provided as a Source Data file.

To further assess how and to what extent temperate viruses affect marine microbes and ecosystems, we inspected their auxiliary metabolic genes (AMGs), integration sites and reprogramming effects. We identified a total of 302 AMGs in 239 marine temperate viruses (Fig. 4a and Supplementary Data 15) and predicted the 3D structures of all AMG-encoded proteins. Notably, 55.0% of them had homologous proteins in the Protein Data Bank (PDB) database with highly similar structures (*Z*-score > 20), indicating that they probably shared similar functions (Supplementary Data 15). Generally, AMGs displayed a niche-dependent distribution pattern, and the AMGs involved in carbohydrate-active enzyme (CAZy) were highly enriched, especially in deep-sea sediments (Fig. 4b). Moreover, the AMGs involved in photosynthesis were exclusively enriched (as expected) in epipelagic seawater, while the transporters encoding AMGs seem to be more preferred in the deep ocean. The CAZy-related AMGs encode diverse enzymes belonging to multiple classes, including glycoside hydrolase (GH, *n* = 11) and glycosyl transferase (GT, *n* = 23) (Fig. 4c). Next, we selected two GH-related AMGs, *vgh5a* and *vgh16c*, to experimentally test whether they encoded enzymatically active proteins. As a result, vGH5A showed hydrolytic activities of β-D-galactosidase, β-D-glucosidase and β-D-fucosidase (Supplementary Fig. 24), in accordance with the functional connectivity of these enzymatic activities^50^. However, vGH16C had no detectable activity under our experimental conditions (Supplementary Data 16). Notably, the predicted 3D structure of the vGH5A protein shared high homology with marine-derived β-glucanase (*Z*-score = 23.8) and soil-derived β-galactosidase (*Z*-score = 23.2) (Supplementary Fig. 24), which can hydrolyse β-(1,3;1,4)-glucans and xyloglucan, respectively^51,52^. These results suggested that AMG *vgh5a* may help microbial hosts hydrolyse polysaccharide substrates in the ocean.

The integration of temperate viruses can substantially change the genomic structure and gene expression of hosts^2^. To examine the preference and potential influences of marine provirus integration, we identified the integration loci of 2794 temperate viruses in host genomes (Supplementary Data 17). Apart from 49.3% of loci located in noncoding regions, 16.5% and 40.9% of the analysed proviruses integrated into transfer RNAs (tRNAs) and protein-coding genes, respectively (Supplementary Fig. 25). The tRNAs Leu, Ser and Arg were integration hotpots. Strikingly, these hotspots were generally consistent with the dominant tRNAs encoded by marine temperate viruses (Supplementary Figs. 25–26 and Data 18), suggesting that integration site selection and the translational compensation of marine proviruses are intrinsically linked. Additionally, we found that the genetic regions related to two-component systems, transporters and transcription factors were preferentially selected for marine temperate virus integration (Supplementary Fig. 25).

**Fig. 5:**
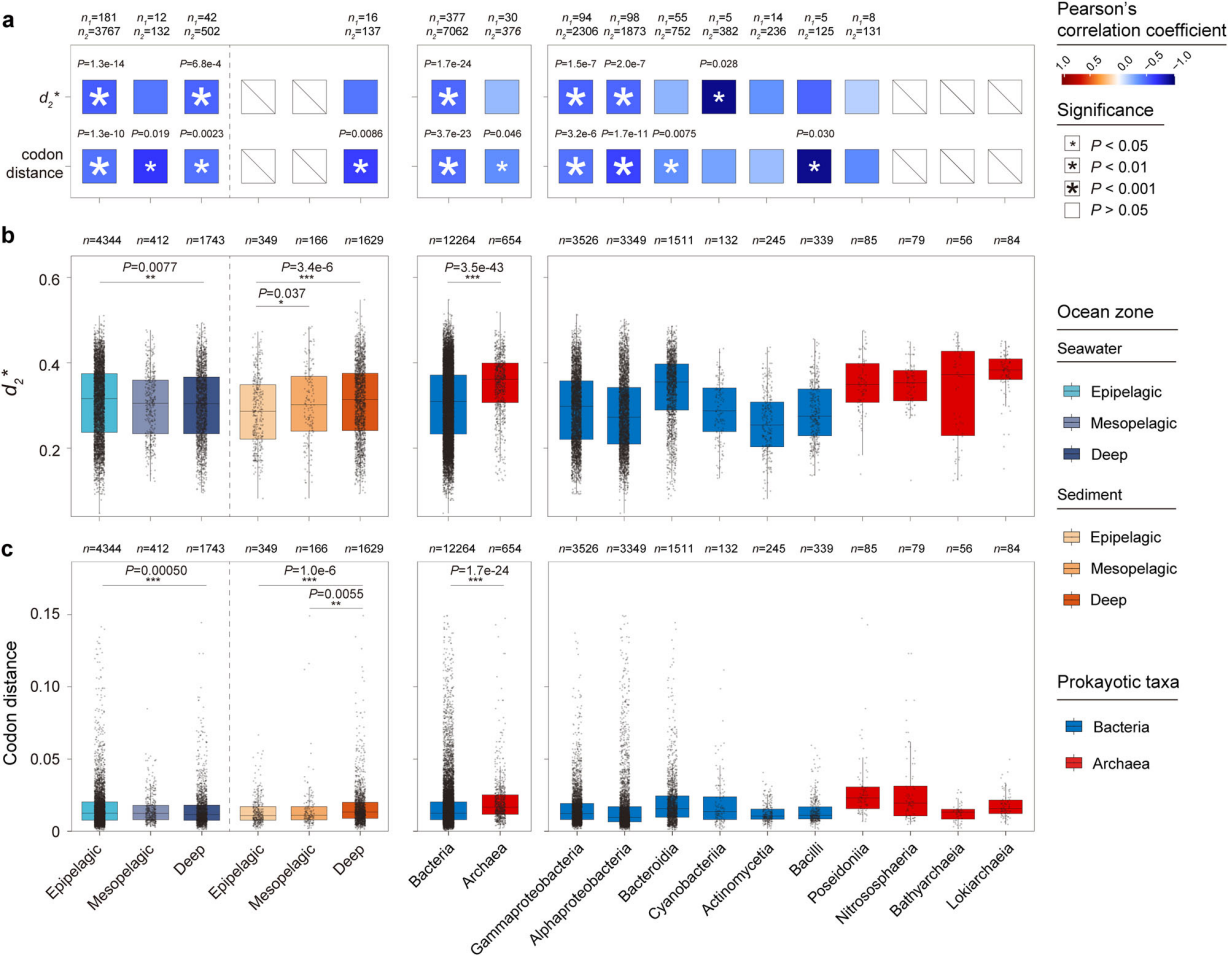
Assessment of marine temperate virus-host complementarity. **a** Correlation between marine temperate virus-host complementarity and lyRs. Pearson’s correlation coefficients between the median *d*_2_*/codon distance and lyRs of genera (with ≥5 genomes) were calculated and are shown by the colour gradient of squares. Significant correlations were determined by two-sided tests and marked by white asterisks and *P*-values are represented by different sizes of asterisks and shown above squares. The slashes indicate statistical unachievability due to the small sample size (number of genera < 5). *n*_*1*_: number of host genera used for calculation of pearson’s correlation; *n*_*2*_: number of host genomes contained in the genera. **b**, **c** Distribution of temperate virus-host nucleotide (**b**) and amino acid complementarity (**c**) in different ocean zones or host taxa. The differences among groups were analysed by the two-sided Wilcoxon rank-sum test, and *P*-values of the significant differences are shown above boxes (other compared groups are shown in Supplementary Data 19). Each box represents the IQR, in which the middle line represents the median. The whiskers extend to 1.5 × IQR, and all contained data are shown as the individual points. The number of virus-host pairs contained in each group is shown at the top of the graphs. Source data are provided as a Source Data file.

Marine virus-induced metabolic reprogramming is related to virus-host genome complementarity^35^. To quantitatively assess this effect in oceans worldwide, we measured oligonucleotide frequency dissimilarity (*d*_2_*) and codon cosine distance between all temperate virus–host pairs and used these variables as indices of nucleotide and amino acid complementarity, respectively (Supplementary Data 19). Remarkably, both indices were negatively correlated with the LyR in different ocean zones and major prokaryotic taxa; the codon distance had more shared correlations, implying high dependency of temperate viruses on host translational machinery (Fig. 5a). We propose virus-host genome complementarity as a general influencing factor of lysogeny prevalence in microbial communities. Subsequently, we compared the two indices among different virus-host groups, and significant differences were observed between the deep and epipelagic ocean, as well as between the bacteria and archaea (Fig. 5b, c). In particular, virus-host complementarity reached the lowest level in the epipelagic seawater and deep-sea sediment groups. These results coincided with the increased divergences in several features between lysogens and nonlysogens in these two marine biomes (Fig. 2 and Supplementary Fig. 12), suggesting that temperate viruses living in these habitats can relatively heavily reprogramme host metabolism and alter host genomic features.

### Marine temperate phages are active and influence the transcriptome of host bacteria

**Fig. 6:**
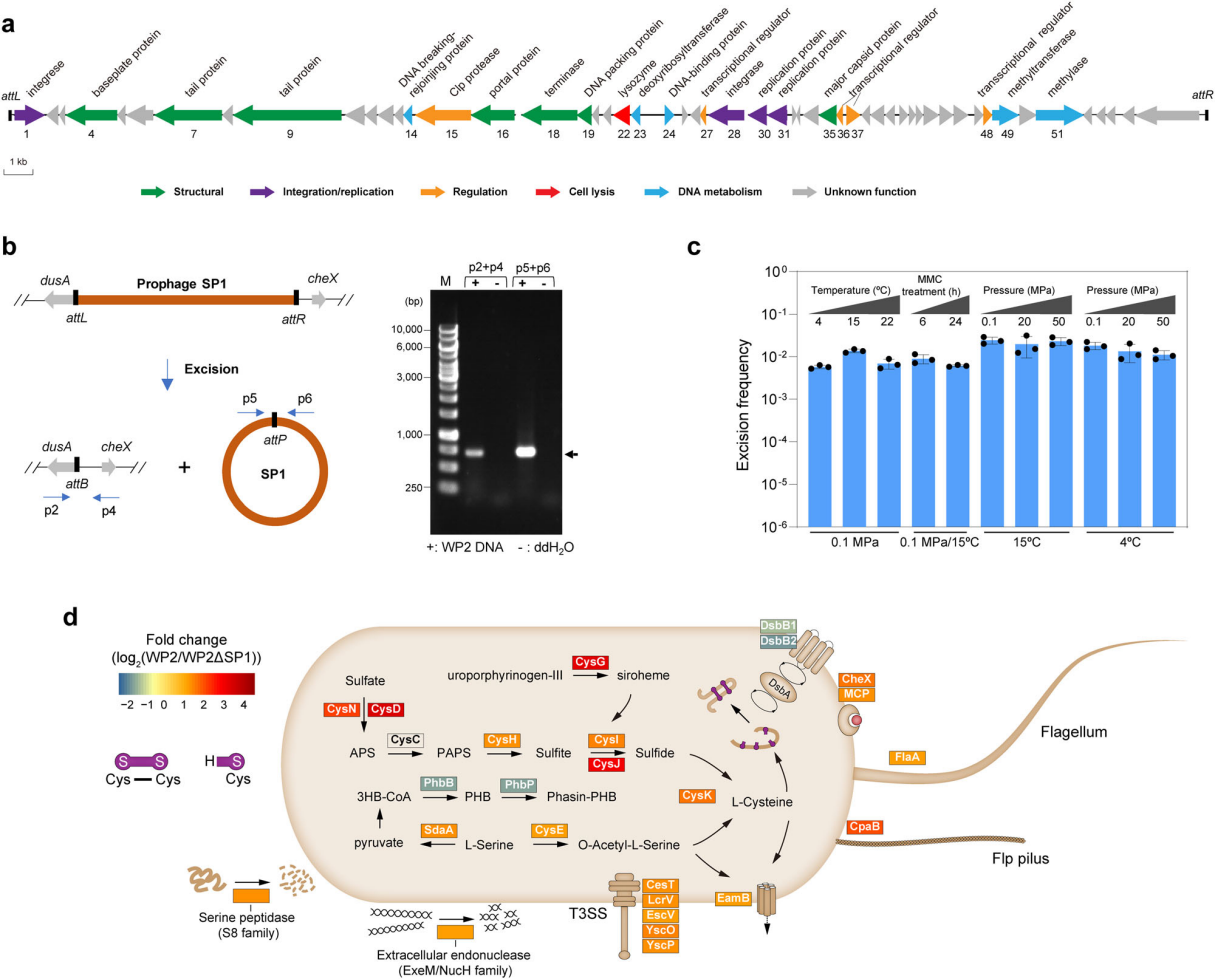
Influence of the marine temperate virus SP1 on the host transcriptome. **a** Genomic map of the prophage SP1 in the marine bacterium *S. psychrophila* WP2 (WP2). The arrows depict the location and direction of predicted proteins on the phage genomes, and the fill colours indicate different functional categories of genes, as indicated in the legend. **b** Verification of SP1 excision by PCR. The left schematic graph shows the process of SP1 excision, in which site-specific recombination occurs through the crossover between *attL* and *attR* sites to generate the SP1-deleted WP2 genome and a circular SP1 genome. The locations of the primer pairs used for verification are also shown. The right panel shows the electrophoresis of PCR products. The primer pairs and template DNA used for PCR are indicated for each lane, and the target bands are marked with an arrow. M, DNA size marker. Image of representative agarose gel from two independent experiments are shown. **c** Excision rates of SP1 in *S. psychrophila* WP2 under different treatments. The data represent the mean ± SD and are based on three biologically independent samples. MMC, Mitomycin C. **d** Graphic display of differentially expressed genes (DEGs) categorized by function in *S. psychrophila* WP2 after SP1 deletion. The transcriptome data represent three biologically independent samples for each strain (WP2 and WP2ΔSP1). Normalized differential expression levels (fold changes) are represented by heatmaps in boxes according to the scale bar (log_2_ scale) from most upregulated to most downregulated. The proteins encoded by the DEGs are shown in each box. Source data are provided as a Source Data file.

To further validate the impacts of temperate viruses on hosts, the marine bacterium *Shewanella psychrophila* WP2 (hereafter referred to as WP2)^53,54^, which was isolated from deep-sea sediment in the western Pacific Ocean and harbours a prophage SP1 (MTVG_4770), was used as a representative temperate virus-host system. The boundary of SP1 was experimentally determined (Supplementary Fig. 27); its genome size is 42,608 bp, and it is integrated into the 5’ terminus of *dusA* and noncoding regions adjacent to the 3’ terminus of *cheX* (Fig. 6a). The excision and circularization of SP1 were verified, indicating its functionality as a complete prophage (Fig. 6b). Intriguingly, SP1 maintained an excision frequency of ~1% under all the tested conditions (Fig. 6c). The frequency is remarkably high compared with that of other prophages (ranging mostly between 10^-7^–10^-4^)^15,17,55–57^, and indicates that SP1 can be actively excised from WP2 genomes in in situ marine habitats.

To investigate the impacts of SP1 on the host, we deleted SP1 from the WP2 genome to obtain the WP2ΔSP1 strain (Supplementary Fig. 27). Although SP1 did not significantly influence the growth of WP2 (Supplementary Fig. 28), it had a significant influence on the transcriptome of WP2 under stimulated in situ environmental conditions (Fig. 6d and Supplementary Fig. 29). Overall, 56 genes were differentially expressed between WP2ΔSP1 and WP2 (Supplementary Data 20). Notably, the transcriptional levels of 7 genes (*cysDNHIGJK*) involved in assimilatory sulfate reduction were significantly upregulated in WP2 compared with WP2ΔSP1 (Fig. 6d). In addition, the genes involved in serine metabolism, including *cysE*, *sdaA*, and *eamB*, were upregulated, and the transcription levels of genes participating in cell motility, the type III secretion system and extracellular hydrolysis were higher in WP2 than in WP2ΔSP1. The 4 downregulated genes were responsible for polyhydroxybutyrate (PHB) synthesis and disulfide bond formation. These results suggest that marine temperate viruses can significantly alter a variety of host physiological activities, including carbon, nitrogen and sulfur metabolism.

Virus-infected cells, termed virocells, exhibit a reprogrammed metabolism and therefore physiologically differ from uninfected cells^58,59^. Specifically, *Pseudoalteromonas* phage PSA-HP1, isolated from surface seawater, drastically alters the central carbon and energy metabolism of its host^35^. In addition, filamentous phage SW1 significantly influences the growth, motility and transcriptome of its deep-sea bacterial host *S. piezotolerans* WP3^19,20^. *Shewanella* strains are ubiquitous in the ocean owing to their versatile metabolism^60,61^. Although SP1 is currently unclassified, this actively excising prophage resembles multiple temperate phages among marine *Shewanella* species (Supplementary Fig. 30), and the recruitment analysis showed that the SP1-like prophages are prevalent in the Pacific Ocean (Supplementary Fig. 31). Therefore, SP1-infected virocells (WP2) and nonvirocells (WP2ΔSP1) are likely to widely coexist in natural marine environments. Our comparative transcriptome analysis of virocells and nonvirocells under simulated benthic marine conditions (high pressure and low temperature) provided experimental evidence for the effects of deep-sea temperate viruses on prokaryotic hosts, data that were previously scarce.

Pioneering global viromic studies have revealed the remarkable abundance, diversity, geographic distribution and ecological roles of virioplankton^25,49,62^. However, the lack of a comprehensive investigation of temperate viruses that reside in marine lysogens hinders a complete understanding of marine viruses. In this study, we systematically revealed the distributions of lysogens and temperate viruses in global oceans (Supplementary Fig. 32), and described the viral diversity as well as underlying genomic features associated with marine lysogens. By examining virus-host interactions, we identified potentially ecologically important tVCs. Combining large-scale data analysis and experimental evidence showing divergences between lysogenic virocells and nonlysogenic cells, we suggest that lysogenic virocells play distinct roles in ecological processes. Taken together, these findings indicate the critical roles of marine temperate viruses in rewiring host metabolism, shaping microbial communities and participating in biogeochemical cycles.

## Methods

### Construction of the Marine Prokaryotic Genome dataset

We collected prokaryotic genomes and accompanying biosample information from the NCBI GenBank (v244) database^28^, GEM^29^ and OTMG^30^ datasets. To screen for marine-derived genomes, corresponding biosamples were preliminarily filtered by keywords, including “marine”, “ocean”, “sea” and “pelagic”. Combined with available references and geographic coordinates, we manually curated the biosamples; only marine-derived biosamples were retained. Next, these candidate marine biosamples were further purified based on the following criteria: (1) excluding biosamples collected at the border between marine and terrestrial environments, including the coast, beach, seashore, estuary and intertidal zone, due to strong terrestrial influences on microbes inhabiting these ecosystems;^63–65^ and (2) excluding host-associated biosamples, such as algae-, coral reef-, eukaryotic plankton- and mammal-associated samples, because the genomes of parasitic/symbiotic microbes are highly influenced by their hosts and differ widely from free-living microbes^66–69^. In addition, the information for each genome-derived sample, including sample type (seawater or sediment) and water depth, was adequately collected and curated, and genomes were classified as epipelagic (0–200 m), mesopelagic (200–1000 m) or deep sea (>1000 m), according to the depth at which they were sampled. If this information was unavailable, the genomes were labelled “not available” (NA).

CheckM (v1.1.3)^70^ was used to evaluate the quality of marine prokaryotic genomes, and only high-quality genomes (completeness > 80% and contamination < 5%) were retained. For species-level descriptions, MUMmer-based^71^ average nucleotide identities (ANIs) among all the genomes were calculated using pyani (v0.2.7);^72^ genomes with > 95% ANIs and 60% alignment length were grouped into prokaryotic operational taxonomic units (OTUs)^29,73^. Moreover, genomes in the same OTU and samples were dereplicated.

Taxonomic classification of all genomes was performed by GTDB-Tk (v1.3.0)^74^ using the “classify_wf” pipeline. Briefly, 120 and 122 marker proteins in the bacterial and archaeal genomes, respectively, were identified, concatenated and aligned^75,76^. Then, the maximum-likelihood placement of a genome was determined in the GTDB-Tk reference tree^77^. Data on the placement in the reference tree, relative evolutionary divergence, and/or ANI to reference genomes were combined to classify a given genome^74^. The phylogenetic tree of marine prokaryotic genomes was constructed by FastTree2 (v2.1.10)^78^ based on the maximum-likelihood algorithm and visualized by iTOL (v6)^79^.

### Construction of the Marine Temperate Virus Genome dataset

First, VirSorter2 (v2.2.2)^80^ was used to predict viral sequences in the curated Marine Prokaryotic Genome dataset with the setting “--include-groups dsDNAphage, ssDNA -min-length 5000 -min-score 0.5”. The mode of searching double-stranded DNA (dsDNA) and single-stranded DNA (ssDNA) viruses was appropriate for recovery of temperate viruses, the minimum score (0.5) was chosen for maximal sensitivity, and the minimum length (5,000 bp) was necessary for the downstream steps. Second, CheckV (v0.8.1)^81^ was used to control the quality of the viral sequences, through steps such as removing nonviral sequences, trimming nonviral genomic regions and evaluating quality. Third, the CheckV-trimmed sequences were passed through VirSorter2 again and formatted to serve as input to DRAM-v (v1.2.4)^82^ for viral annotation. Retained viral sequences were curated according to the widely recognized empirical screening criteria based on the information of viral and host gene counts, score, hallmark gene counts and sequence length^83^. The sequences requiring manual checking were further curated according to summarized criteria based on detailed viral annotation from DRAM-v^82^. Specifically, the viral sequences > 200 kb were screened based on a highly confident VirSorter2 score (cut-off of 0.9) and strictly curated, resulting in 25 sequences being discarded, because long sequences with lower scores frequently contained dense nonviral proteins and are normally considered false positives^80,83^. In addition, we excluded 15 viral sequences annotated as φX174 that were commonly used as quality control in Illumina sequencing^84^.

### Assessment of the prevalence of lysogens

Lysogeny ratios (LyRs) for each taxon and environment were calculated to determine the prevalence of lysogens. The LyRs in different prokaryotic taxa were examined at the superkingdom, class, and genus levels (Supplementary Data 7). In the statistical analyses, only genera with ≥5 genomes and classes with ≥20 genomes in the MPGD were analysed. Polylysogeny and coinfection of temperate viruses were defined as the detection of multiple distinct temperate viruses in a single prokaryotic host^84^. However, the frequency of polylysogeny can be overestimated if a single viral genome is misassembled and/or split into several contigs. To avoid overestimation, we adopted rigorous criteria to determine polylysogeny and coinfection, according to the previous study^84^. Briefly, the large unit of the terminase (TerL) was considered as a single-copy marker protein (SCMP) in Caudovirales and other unclassified viruses^84^. In addition, the zonular occludens toxin (Zot) was considered as a SCMP in Inovirales^85^. Only viral genomes containing SCMPs were included when calculating the polylysogeny ratio.

### Viral clustering, taxonomic assignment and network analysis

Prodigal (v2.6.3)^75^, with default parameters, was used to determine the open reading frames (ORFs) of temperate viral genomes. All the viral proteins were clustered by CD-HIT (v4.8.1)^86^ using 60% identity, 80% coverage of the shorter sequence and the recommended parameter of ‘-g 1 -n 4 -d 0’^24^. They were also coclustered with viral proteins of the IMG/VR provirus database (v3)^31^, using the same parameters, to enable protein family comparison.

We used pyani (v0.2.7)^72^ to group temperate viral genomes sharing 95% MUMmer-based^71^ ANIs with at least 85% length coverage into temperate viral OTUs (tvOTUs), at approximately the species level^87^. They were also coclustered with genomes from the IMG/VR provirus database^31^ with the same parameters for vOTU comparison. Next, viruses ≥10 kb were clustered into temperate viral clusters (tVCs) using vConTACT2 (v2.0);^88^ in brief, all-to-all BLASTp, similarity calculation and Markov clustering were performed with the default parameters^88^. The reference viral genomes provided by vConTACT2 were coclustered with query genomes for taxonomic prediction. A query genome was assigned the taxonomy of clustered reference genomes, in which the taxonomy occupying the highest proportion was chosen. To supplement this analysis, the taxonomy of viral genomes not assigned in the vCONTACT2 pipeline and < 10 kb in size was further predicted for taxonomy by a “majority-rules approach”^49^. In detail, all of their proteins were subjected to Diamond (v0.9.14.115)^89^ for BLASTp alignment against RefSeq Virus (v207);^90^ a viral genome was considered to belong to a viral family if ≥50% of the proteins were assigned to that family with a bitscore ≥50. The host range of a tVC was calculated as the number of different host taxa its included viruses infected.

To conservatively evaluate host ranges, we further clustered viruses into temperate viral genera (tVG) using VIRIDIC^41^ based on a nucleotide identity cut-off of 70%, according to the ICTV standard^91^. The tVG with cross-class/phylum/superkingdom host range were further checked by host prediction based on virus-host homology of CRISPR spacer and nucleotide sequence, which were previously demonstrated to show high precision^92^. Prokaryotic genomes from the RefSeq database (Release 206, *n* = 216,865) and the MPGD (*n* = 12,080) were used for the prediction. The CRISPRs of prokaryotic genomes were predicted using the CRISPR Recognition Tool (CRT)^93^ with optimized parameters as previously described^94^, and CRISPR clusters with less than three spacers were discarded. The CRISPR spacers were aligned against the viral contigs using BLASTn and matches with 100% coverage and <2 mismatches were retained^95^. For nucleotide homology, viral contigs were aligned against prokaryotic genomes using BLASTn with the cutoff of bitscore >50, *e*-value < 1e-3, identity > 70% and match length > 2500 bp^96^.

The virus-host interaction network analysis was based on the number of infections by a given tVC in a given prokaryotic genus, which was calculated as the number of temperate viral genomes in a given tVC present in a given host genus. In the network, prokaryotic genera and tVCs were represented as distinct nodes, and the number of infections was represented as shared edges. Similarly, the coinfection network analysis was based on the number of coinfections with tVCs, and only tVCs carrying SCMPs were included. In this network, tVCs were represented as nodes, and the number of coinfections of two tVCs was represented as shared edges. Protein sharing network analysis of SAR11 viruses was performed by vConTACT2^88^. All of the networks were visualized and analysed by Cytoscape (v3.7.0)^97^ using the edge-weighted spring-embedded model.

### Characterization of genomic features and maximal growth rates

Protein coding density was calculated as the number of predicted proteins per kb genome. Codon relative frequencies were calculated using the “uco” function in the R package *seqinr*^35^. The script “get_gc_and_narsc.py” was used to calculate the GC content and nitrogen/carbon atoms per residue side chain (N/C-ARSC) of the genomes^98^. For analysis of microbial genomic features, we excluded temperate viral sequences from microbial genomes and calculated the genomic features of these “pure” host genomes. The maximal growth rates of prokaryotes were estimated by the R package gRodon (v1.0) based on genome-wide codon usage statistics^99^. The genes encoding ribosomal proteins, which are typically highly expressed in prokaryotes, were identified by BLASTp alignment against bacterial ribosomal proteins (br01610) in the Kyoto Encyclopedia of Genes and Genomes (KEGG) database^100^ with *e*-value < 1e-10, identity > 30% and coverage > 50%. The Minimal doubling time (MDT) was calculated using the “predictGrowth” function in the gRodon package, with data from whole genes and genes encoding ribosomal proteins as the input. If a genome was incomplete, the function was set to the “partial” mode. As fast-growing copiotrophs in the ocean normally exhibit a maximum growth rate of >1 d^-1^, and MDT prediction appeared to be accurate only up to 5 h in practice^99,101^, only prokaryotes with a predicted MDT < 5 h were considered fast growers in the study.

The nucleotide and amino acid dissimilarity between each virus-host pair were calculated using the method previously reported^35^. Briefly, oligonucleotide frequency dissimilarity *d*_2_* at *k* = 6 was adopted to determine the nucleotide distance between a virus and host, using VirHostMatcher^102^ with default parameters, and a cosine distance between the codon frequency vectors of the virus and host (Dc(phage, host)) was used to describe their amino acid dissimilarity^35^.

### Identification of putative auxiliary metabolic genes (AMGs)

All of the marine viral genomes were annotated by the DRAM-v workflow^82^. Then, each viral gene was assigned metabolic flags and an auxiliary score, determined by its location on the viral sequence relative to other viral hallmarks or viral-like genes identified by VirSorter^103^. According to the usage guidelines, only viral genes with a metabolic flag’M’ and a confidence score of 1–3 were considered as candidate AMGs^82^. To further screen high-confidence AMGs, we manually curated the candidate AMGs to retain those with unambiguous virus-like genomic contexts, referring to conservative criteria reported previously^96^. Specifically, a conserved set of AMGs was required to have viral structural genes, terminases or integrases in genomic regions both downstream and upstream of the gene. In addition, we manually deleted the candidate AMGs that are probably involved in viral functions, including DNA methylase- and nucleotide metabolism-related genes. Gene annotations of viral genomes containing AMGs are displayed in Supplementary Data 15 in which the putative AMGs, viral structural genes, terminases and integrases are marked. In AMG distribution analyses, a relative abundance of an AMG category was calculated as the average number of included AMGs carried by per viral genome, which was dereplicated according to their gene IDs.

### Analysis of integration sites of marine temperate viruses

After a temperate virus integrates into a host genome, the provirus usually ends with two attachment sites, *attL* and *attR*, which display as short repeated sequences^104^. To ensure reliability, only temperate viral genomes with both two flanking host sequences which contained ≥5 ORFs were included in the integration site analysis. In line with previous studies^105,106^, we scanned perfect repeated sequences within 1000 bp inside the viral boundaries, as determined by VirSorter2 and/or CheckV, and the length of *att* sites ranged from 2–145 bp, with a bias toward longer repeats. The host sequences upstream of *attL* and downstream of *attR* were reconnected to recover the integration loci, and then subjected to a BLASTx^89^ alignment against the KEGG database^107^ online with an e-value cut-off of 1e-10. In addition, tRNAscan-SE (v2.0.3)^108^ was used to predict tRNA in the integration loci, as well as in all temperate viral genomes. The “-B” and “-A” modes were combined for the bacterial and archaeal tRNA prediction. The context of the integration loci was examined by analysing 5 flanking host genes; these genes were annotated by BLASTp^89^ alignment against the KEGG database^107^ online with an e-value cut-off of 1e-10. Only temperate viral genomes with ≥1 flanking host sequences which contained ≥5 ORFs were included in this analysis.

### Statistical analysis

The two-sided Wilcoxon rank-sum test^109^ was adopted to evaluate all the significance of differences between groups using the Python function “ranksums” from Scipy (v1.5.0)^110^. Pearson correlation analysis^111^ was used to determine the relationship between LyRs and genomic features at the genus level via the Python function “pearsonr” (two-sided test) from SciPy (v1.5.0);^110^ only the genera with ≥5 genomes were included in this analysis.

### Read recruitment analysis

Read recruitment of marine temperate viruses in the Pacific Ocean Virome (POV)^24^ was performed by BLASTn alignment as previously reported^112^, using an e-value ≤ 10^-5^, identity ≥ 95% and hits >50 bp. The relative abundance was calculated as the number of mapped reads per kilobase of temperate viral genome per billion reads in each metagenome. Recruitment analysis of SP1-like viruses was performed by BLASTn with an e-value cut-off of ≤ 10^−3^ against metagenomic raw reads from the POV samples with water depths <1000 m and ≥1000 m.

### Functional characterization of viral glycoside hydrolases

The *vgh5A* gene (MTVG_4775_21) encoding a viral glycoside hydrolase was synthetized and cloned into the pET28a plasmid (Saiheng Biotechnology, Shanghai, China), and the recombinant plasmid was transformed into *E. coli* BL21 (DE3) cells. The transformant was selected on LB medium containing kanamycin and confirmed by PCR and DNA sequencing. *E. coli* BL21 (DE3) cells containing the vGH5A expression vector were grown in LB broth with 50 μg/ml kanamycin at 37 °C. vGH5A expression was induced by the addition of IPTG (0.2 mM) when the OD_600_ reached 0.6, and the culture was then incubated at 15 °C for 16 h. The cells were collected by centrifugation, resuspended in binding buffer [500 mM NaCl, 10% glycerol, and 20 mM Tris–HCl (pH 8.0)], and sonicated on ice. The cell extract was clarified by centrifugation at 10,000 × g for 45 min at 4 °C. Ni-NTA Sefinose (TM) Resin 6FF (Sangon Biotech, Shanghai, China) was used to purify His-tagged vGH5A according to the manufacturer’s instructions. The protein was eluted in elution buffer [500 mM NaCl, 10% (v/v) glycerol, 300 mM imidazole, and 20 mM Tris–HCl (pH 8.0)]. The purity of the protein was confirmed by SDS-PAGE (12% acrylamide) with visualization using Coomassie Brilliant Blue R-250, and the concentration of the purified protein was determined using a Nanodrop 2000 spectrophotometer (Thermo Scientific, Waltham, USA).

The enzyme activity of glycoside hydrolysis was determined using the p-nitrophenyl (*p*NP) and 3,5-dinitrosalicylic acid (DNS) methods as previously described^113,114^. For the pNP method, *p*NP-β-D-xylopyranoside (pNP-βXyl) (Haohong Biomedical Technology, Shanghai, China), *p*NP -β-D-mannopyranoside (pNP-βMan) (Macklin Biochemical Technology, Shanghai, China), *p*NP -β-D-galactopyranoside (pNP-βGal) (Macklin), *p*NP -β-D-fucopyranoside (pNP-βFuc) (Macklin), *p*NP -α-L-arabinofuranoside (pNP-αAra) (Solarbio Science & Technology, Beijing, China) and *p*NP -β-D-glucopyranoside (pNP-βGlc) (Biosynthesis Biotechnology, Beijing, China) were used as substrates, and the enzymatic activity measurement was performed in final volumes of 210 μl of 50 mM sodium phosphate buffer (pH 7.0) containing 1 mM p-nitrophenyl-glycosides and 10 μg recombinant vGH5A. After incubation at 50 °C for 60 min, 20 μl of Na_2_CO_3_ (2 M) was added to stop the reaction and stabilize the chromophore in its anionic 4-nitrophenolate form. The enzyme activity was determined colorimetrically at 410 nm by measuring the released p-nitrophenol using a Synergy H1 microplate reader (BioTek, Winooski, USA). For the DNS method, polysaccharides including barley β-glucan (Macklin), carboxymethylcellulose (Titan Biotech, Shanghai, China), lichenan (Megazyme, Bray, Ireland), xyloglucan (Megazyme), and extracts of sargassum (Ruiyuanze Biotech, Gansu, China), porphyra (Ruiyuanze), kelp (Ruiyuanze), bladderwrack (Ruiyuanze), and locust bean gum (Macklin) were used as substrates. The enzymatic activity measurement was performed in final volumes of 50 μl of 50 mM sodium citrate buffer (pH 7.0) containing 1% (w/v) polysaccharide substrate and 10 μg recombinant vGH5A. Following incubation at 30 °C for 60 min, the reaction mixture was heated at 100 °C for 5 min to stop the reaction. and stabilize the chromophore in its anionic 4-nitrophenolate form. The enzyme activity was determined colorimetrically at 540 nm by measuring the liberated glucose using a Synergy H1 microplate reader (BioTek, Winooski, USA). For all the enzymatic assays, the reaction mixtures without enzyme and with the heat-inactivated enzyme were used as the blank and the negative control, respectively. The same procedures were used for the functional characterization of another viral glycoside hydrolase vGH16C (MTVG_6627_10), except that *vgh16C* was cloned into the pMAL-c2x vector (Takara Bio, Dalian, China) using the ClonExpress II One Step Cloning Kit (Vazyme Biotech, Nanjing, China), and the recombinant plasmid was transformed into *E. coli* Rosseta (DE3) cells.

AlphaFold (v2.1)^115^ was used to predict the 3D structures of the AMGs-encoded proteins. Distance matrix alignment (DALI, online version, http://ekhidna2.biocenter.helsinki.fi/dali/)^116^ was used to calculate the structure similarity between AMG-encoded proteins and proteins in the Protein Data Bank (PDB) database^117^. The structure of vGH5A was visualized by PyMOL (v2.0)^118^.

### Culture conditions and growth assays

All bacterial strains and plasmids used in this study are listed in Supplementary Table 1. The *Shewanella* strains were cultured in modified 2216E marine media (2216E) (5 g/l tryptone, 1 g/l yeast extract, 0.1 g/l FePO_4_, 34 g/l NaCl) and shook at 220 rpm at 4 °C and 15 °C; stainless steel pressure vessels were used for cultivation at high hydrostatic pressure. *Escherichia coli* strains were incubated in lysogeny broth (LB) media (10 g/l tryptone, 5 g/l yeast extract, 10 g/l NaCl) supplemented with 50 μg/ml DL-α, ε-diaminopimelic acid (DAP) at 37 °C. For solid media, agar-A (Bio Basic Inc., Ontario, Canada) was added at 1.5% (w/v). The antibiotic chloramphenicol (Cm) (Sigma, St. Louis, USA) was added to the media at final concentrations of 25 μg/ml and 12.5 μg/ml for *E. coli* and *Shewanella*, respectively, when needed. The growth of the *Shewanella* and *E. coli* strains was determined using turbidity measurements at 600 nm with a spectrophotometer (UV-2550, Shimadzu, Kyoto, Japan).

### Construction of the SP1 deletion strain

The SP1 prophage deletion mutant was constructed by a recombination knock-out method^19^. Briefly, the upstream and downstream fragments flanking both ends of SP1 were amplified with PCR primer pairs (Supplementary Table 2) which were synthesized by Sangon Biotech (Shanghai, China). These two fragments were used as templates in a second fusion PCR, resulting in a fusion fragment flanking the boundary of SP1. Then, the PCR product was cloned into the suicide plasmid pRE112, yielding pRE112-SP1. This plasmid was transformed into *E. coli* WM3064 and then into WP2 by two-parent conjugation. The transconjugant was selected by chloramphenicol resistance and verified by PCR. The WP2 strain with pRE112-SP1 inserted into the chromosome was plated on 2216E agar medium supplemented with 10% sucrose. A successful prophage deletion mutant was screened for and confirmed by PCR and DNA sequencing.

### Determination of the SP1 excision frequency

Quantitative PCR (qPCR) was used to determine the frequency of prophage excision under different conditions as previously described^56^. Specifically, the number of WP2 genomes was determined based on the single-copy reference gene *rho*. The number of genomes devoid of SP1 prophage was determined using primers (Sangon Biotech, Shanghai, China) flanking the prophage, which only produce PCR products when the prophage is removed.

### RNA isolation and RT-qPCR

The *S. psychrophila* WP2 strains were inoculated into 2216E media, after which the culture was collected and immediately frozen in liquid nitrogen when the cells reached the exponential phase. Total RNA was isolated with a TRI reagent-RNA isolation kit (Molecular Research Center, Cincinnati, USA). The RNA samples were treated with DNase I at 37 °C for 1 h to remove DNA contamination, and then the purified RNA was reverse transcribed to complementary DNA (cDNA) by a RevertAid First Strand cDNA Synthesis Kit (Fermentas, Maryland, USA). The primer pairs used to amplify the selected genes for RT-qPCR were designed using Primer Express software (v3.0.1) (Applied Biosystems, CA, USA). PCR cycling was conducted using a StepOnePlus real-time PCR system (Thermo Fisher Scientific) in 20 μl reaction mixtures that included 1 × SYBR Green I Universal PCR Master Mix (Thermo Fisher Scientific), 0.5 μM of each primer, and 1 μl of cDNA template.

### Transcriptome analysis

Strand-specific transcriptome sequencing was performed at Magigene Biotechnology Co., Ltd. (Guangdong, China). First, ribosomal RNA (rRNA) was removed using an Epicentre Ribo-Zero rRNA Removal Kit (Epicentre, Madison, WI, USA), and a cDNA library was prepared with a NEBNext Ultra II Directional RNA Library Prep Kit for Illumina (NEB, Ipswich, MA, USA) according to the manufacturer’s instructions^119^. The initial quantification of the library was carried out using a Qubit Fluorometer (Life Technologies, Carlsbad, CA, USA), and the insertion fragment size of the library was determined with an Agilent 2100 Bioanalyser (Agilent Technologies, Palo Alto, CA, USA). The effective concentration of the library was accurately quantified via qPCR (effective concentration > 2 nM). The different libraries were pooled together in a flow cell according to the effective concentration and the target offline data volume. After clustering, the Illumina HiSeq sequencing platform (Illumina, San Diego, USA) was used for paired-end sequencing. The raw data were filtered and evaluated by fastp software (v0.19.7)^120^, after which the clean reads were mapped to the *S. psychrophila* WP2 genome (NZ_CP014782.1) by HISAT software (v2.1.0)^121^. RSEM (v1.3.1)^122^ was used to calculate the read counts per sample, and the sequencing results were evaluated in terms of quality, alignment, saturation, and distribution of reads on the reference genome by DEGseq (v1.36.0)^123^. Gene expression was calculated by the number of reads mapped to each gene using the fragments per kilobase per million mapped reads (FPKM) method^124^ and analysed by edgeR (v3.20.2)^125^. The DEGs were identified according to the following standards: a false discovery rate (FDR) <0.05 and an FPKM fold change ≥2 between two samples. For each strain, three biologically independent samples were used for the RNA-seq analysis. The transcriptomic data were validated via RT-qPCR analysis, and a high correlation coefficient (*R*^*2*^ = 0.9593) was revealed (Supplementary Fig. 29), indicating that the transcriptomic data were reliable and could be used for follow-up analysis.

### Reporting summary

Further information on research design is available in the Nature Portfolio Reporting Summary linked to this article.

### Supplementary information

Supplementary Information

Peer Review File

Description of Additional Supplementary Files

Supplementary Data 1

Supplementary Data 2

Supplementary Data 3

Supplementary Data 4

Supplementary Data 5

Supplementary Data 6

Supplementary Data 7

Supplementary Data 8

Supplementary Data 9

Supplementary Data 10

Supplementary Data 11

Supplementary Data 12

Supplementary Data 13

Supplementary Data 14

Supplementary Data 15

Supplementary Data 16

Supplementary Data 17

Supplementary Data 18

Supplementary Data 19

Supplementary Data 20

Supplementary Data 21

Reporting Summary

### Source data

Source Data

## Data Availability

All assembled prokaryotic genomes (*n* = 12,080) used in this study were collected from publicly available databases including the NCBI GenBank (v244)^28^, GEM^29^ and OTMG^30^. The nucleotide sequences of marine temperate viral genomes (*n* = 12,918) were deposited in the Supplementary Data 21. The transcriptomic data from the current study was deposited in the NCBI SRA under project ID PRJNA1014337. Source data are provided with this paper.

## Code availability

All custom scripts, network and phylogenetic tree files used in this study are available on Github at https://github.com/kaixinbei132/Marine-temperate-viruses and the Zenodo repository (10.5281/zenodo.8289908).
