# Supplementary material for: A systematic analysis of marine lysogens and proviruses
Source: Nat Commun. 2023 Sep 27;14:6013. doi: 10.1038/s41467-023-41699-4 (PMC10533544; doi:10.1038/s41467-023-41699-4)
Supplement: Supplementary file 1 — Supplementary Information [file 41467_2023_41699_MOESM1_ESM.pdf]

Supplementary Information For

A systematic analysis of marine lysogens and prophages

Yi Yi[1], Shunzhang Liu[1], Yali Hao[1, 2], Qingyang Sun[1], Xinjuan Lei[1, 2], Yecheng Wang[1], Jiahua Wang[1], Mujie Zhang[1, 2], Shan Tang[1, 2], Qingxue Tang[1], Yue Zhang[1], Xipeng Liu[1, 2], Yinzhao Wang[1, 2], Xiang Xiao[1, 2, 3], Huahua Jian[1, 2]*

Affiliations:

[1]State Key Laboratory of Microbial Metabolism, Joint International Research Laboratory of Metabolic & Development Sciences, School of Life Sciences and Biotechnology, Shanghai Jiao Tong University, Shanghai, China

[2]Yazhou Bay Institute of Deepsea Sci-Tech, Shanghai Jiao Tong University, Sanya, China

[3]Southern Marine Science and Engineering Guangdong Laboratory (Zhuhai), Zhuhai, China

*Corresponding author. E-mail: jiandy@sjtu.edu.cn.

Supplementary Figs. S1-S32

Supplementary Tables S1 and S2

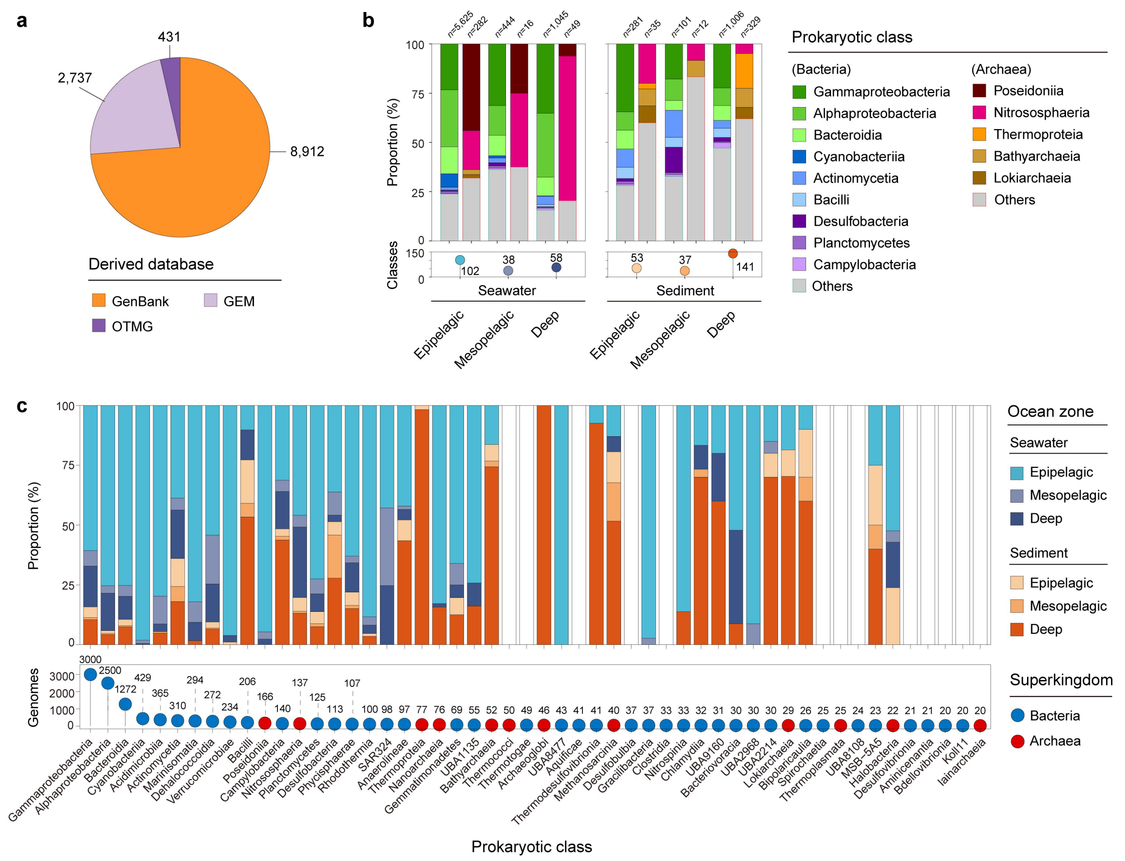

Fig. S1| Composition of the Marine Prokaryotic Genome dataset (MPGD). a, The source databases of the MPGD. GEM: Genomes from Earth's Microbiomes; OTMG: Oceanic Trench Microbial Genome. **b**, Taxonomic composition of prokaryotes in different ocean zone groupings. The number of genomes contained in each ocean zone is shown above each column. The number of classes is shown next to the circles. **c**, Distribution of prokaryotic classes in different ocean zones. The classes without environmental information are shown as uncoloured bars. Only classes with ≥ 20 genomes are displayed, and the number of genomes contained in each class is shown above the filled circles.

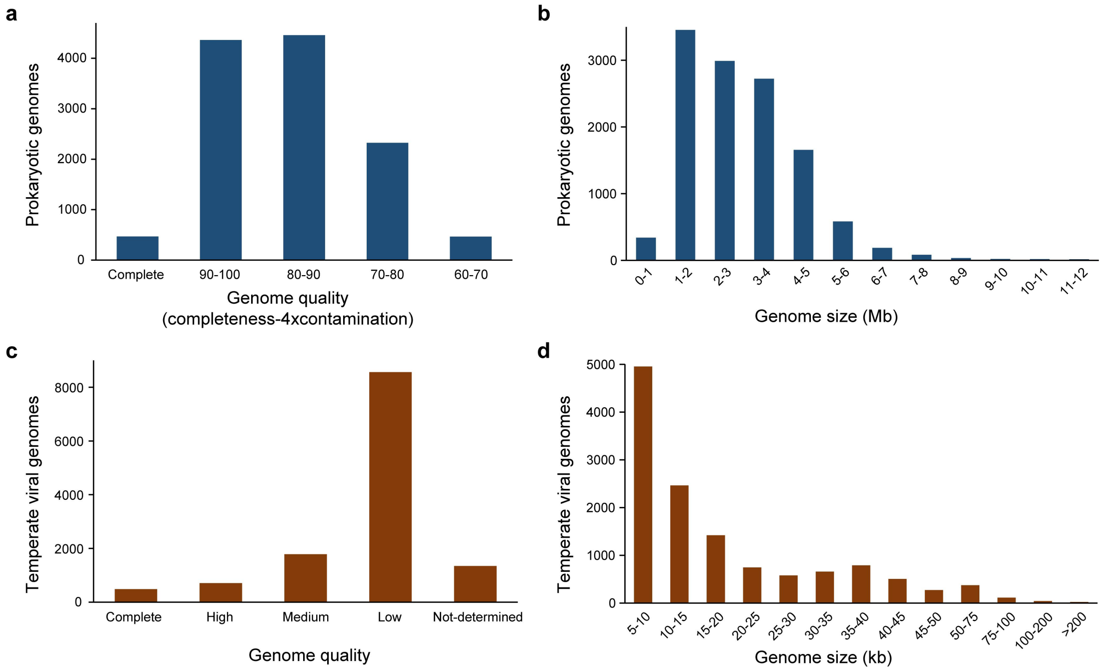

Fig. S2| Genome quality evaluation and size distribution of the MPGD (a, b) and MTVGD (c, d). The genome quality of prokaryotic and viral genomes was evaluated by CheckM[1] and CheckV[2], respectively.

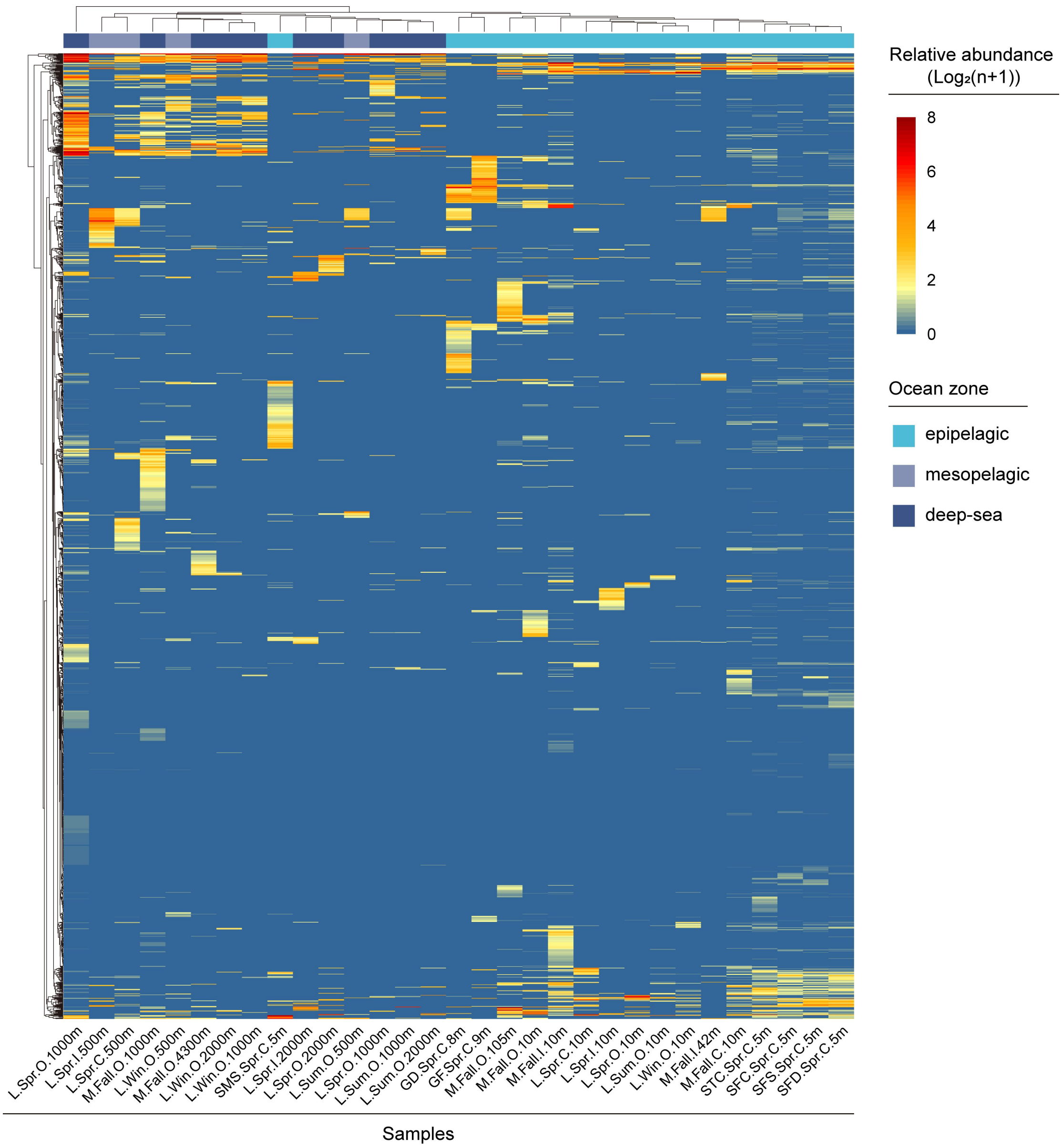

Fig. S3| Recruitment analysis of genomes from the MTVGD in the Pacific Ocean virome (POV). The heatmap shows the relative abundance of 1915 temperate viral genomes in the POV dataset[3], which is hierarchically clustered both by samples and viral genomes. Read recruitment was performed by BLASTn alignment using an e-value ≤ 10^{-5}, identity ≥ 95% and hits > 50 bp. The relative abundance was calculated as the number of mapped reads in per kilobase of temperate viral genomes from per billion of reads in each metagenome.

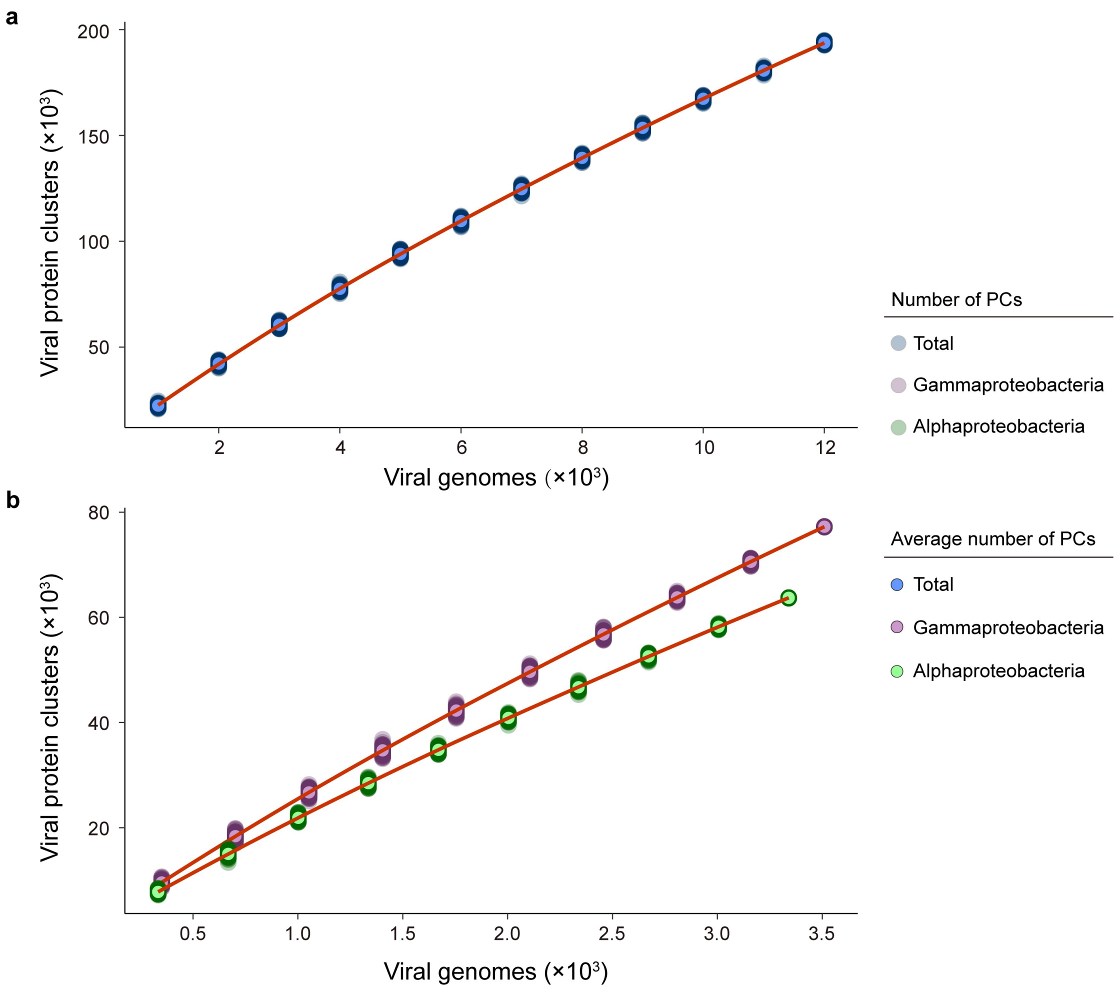

Fig. S4| Saturation analysis of viral protein clusters in genomes in the MTVGD (a), and in temperate viruses that infect Gammaproteobacteria (b, purple points) and Alphaproteobacteria (b, green points). The dark points represent the numbers of protein clusters (PCs) in viral genomes after 100 random samples, and the light points show the average number of PCs. The red lines refer to the fitting lines based on the average values.

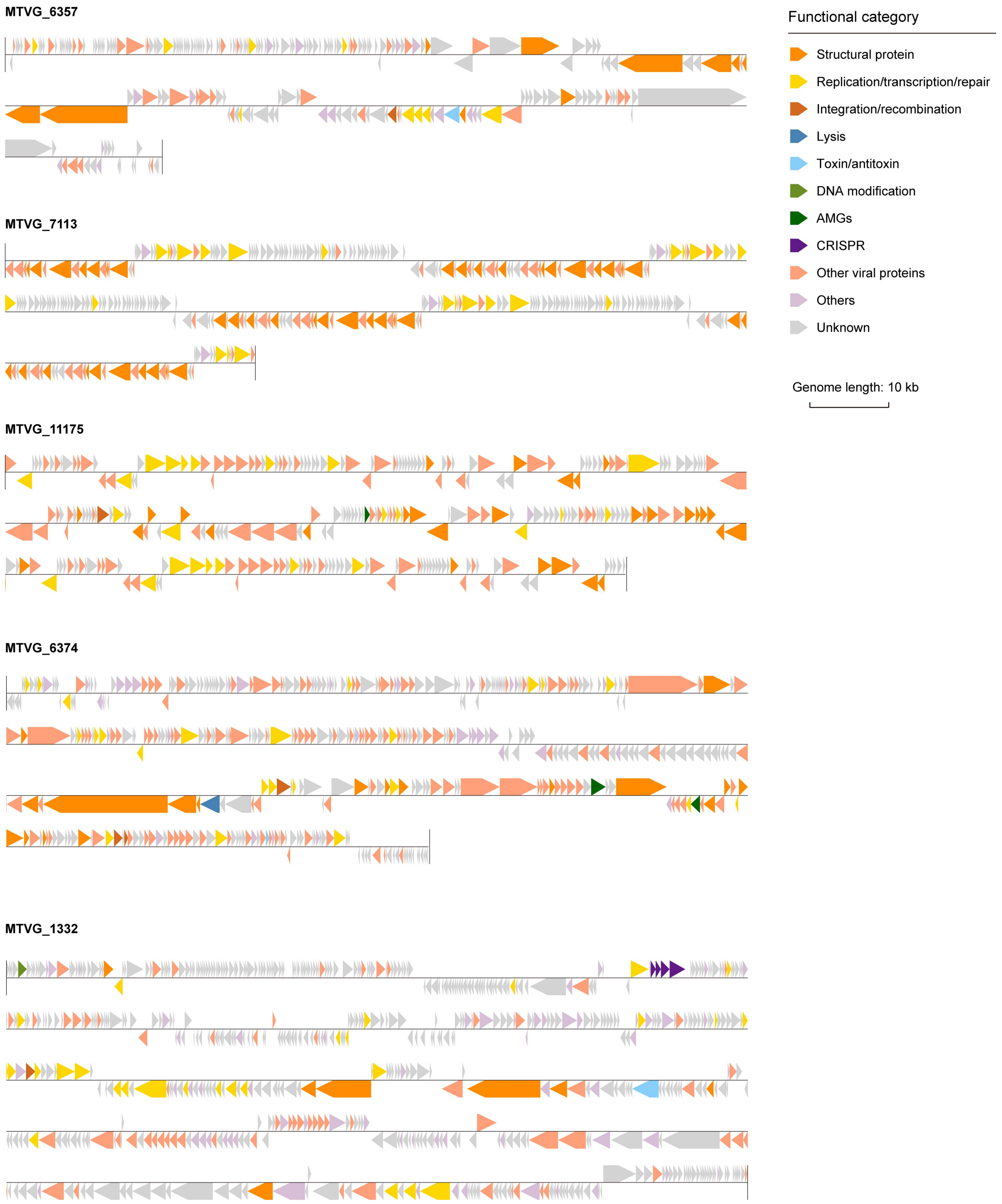

Fig. S5| Genome maps of representative huge temperate phages in the MTVGD. The arrows depict the location and direction of predicted proteins on the viral genomes, and the fill colours indicate different functional categories of genes, as shown in the legend.

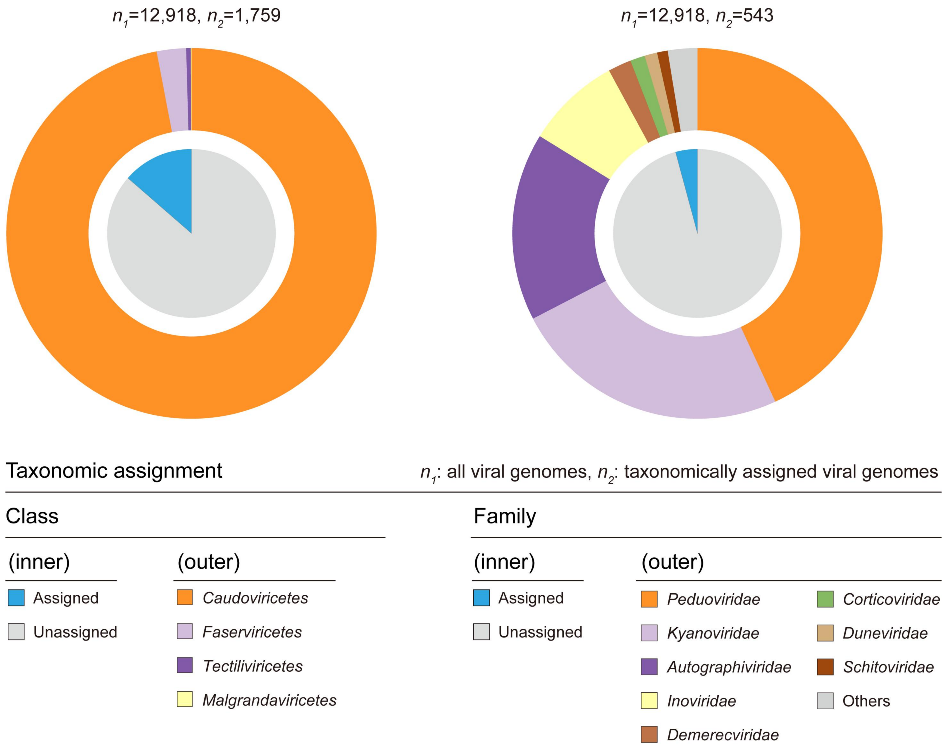

Fig. S6| Taxonomic assignment of marine temperate viruses. The taxonomy prediction combines the results from vCONTACT2 and a "majority-rules approach" (see the Methods section for a detailed description). The left and right charts show taxonomic compositions at the class (left) and family (right) levels, respectively. The inner pie charts indicate the percentages of viral genomes that have predicted taxonomy, and the outer pie charts show the percentages of viral taxa, considering only viruses with predicted taxonomy.

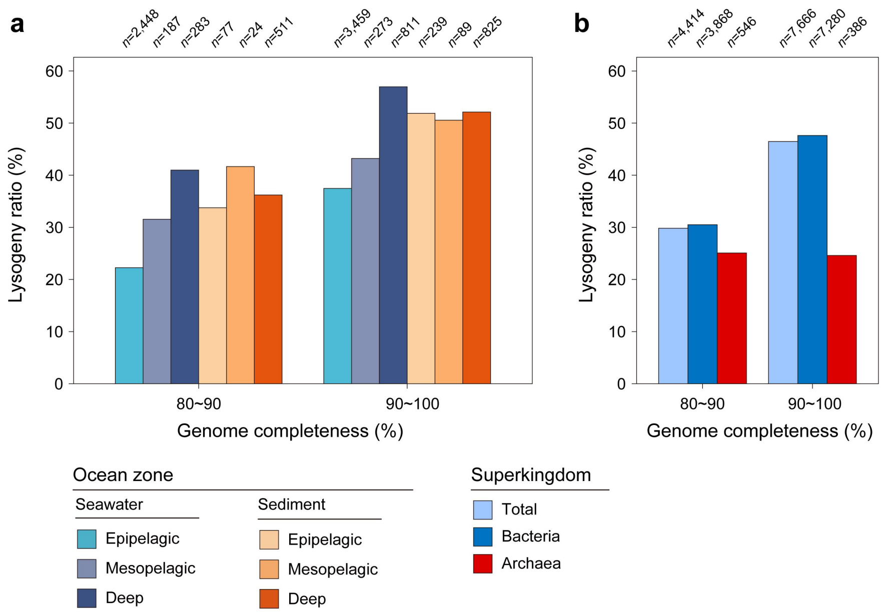

Fig. S7| Lysogeny ratios of marine prokaryotic genomes in different ranges of completeness. The analyses were performed for genomes from different ocean zones (**a**) and superkingdoms (**b**). The number of genomes contained in each group is shown above each column.

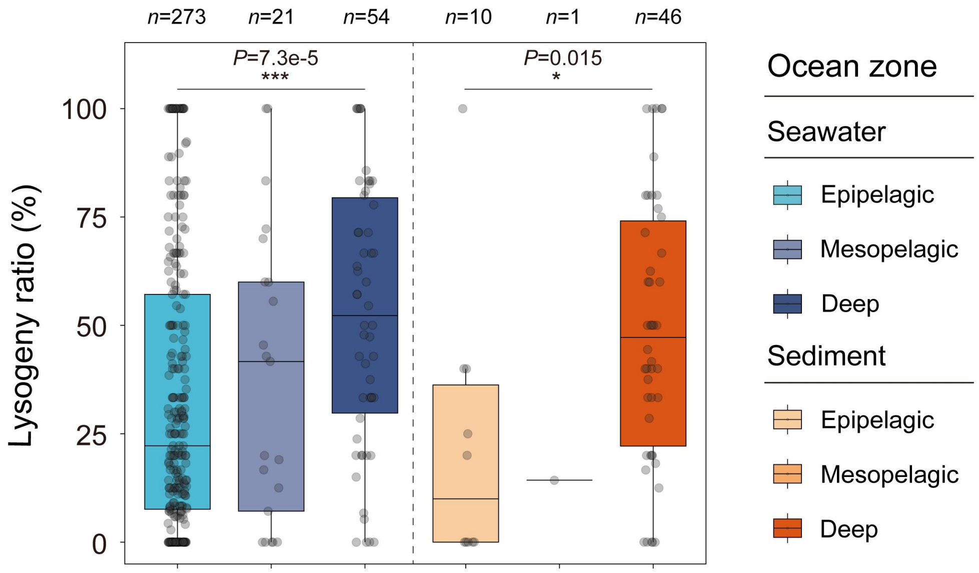

Fig. S8| Comparison of LyRs at the prokaryotic genus level in different ocean zones. In statistical analysis, only genera with ≥ 5 genomes were used in the calculation of LyRs. The number of genera contained in each ocean zone is shown at the top of the plot. The differences were analysed by the two-sided Wilcoxon rank-sum test and *P* values of significant differences are shown above the boxes. Each box represents the interquartile range (IQR), in which the middle line represents the median. The whiskers extend to 1.5×IQR, and all contained data are shown as the individual points.

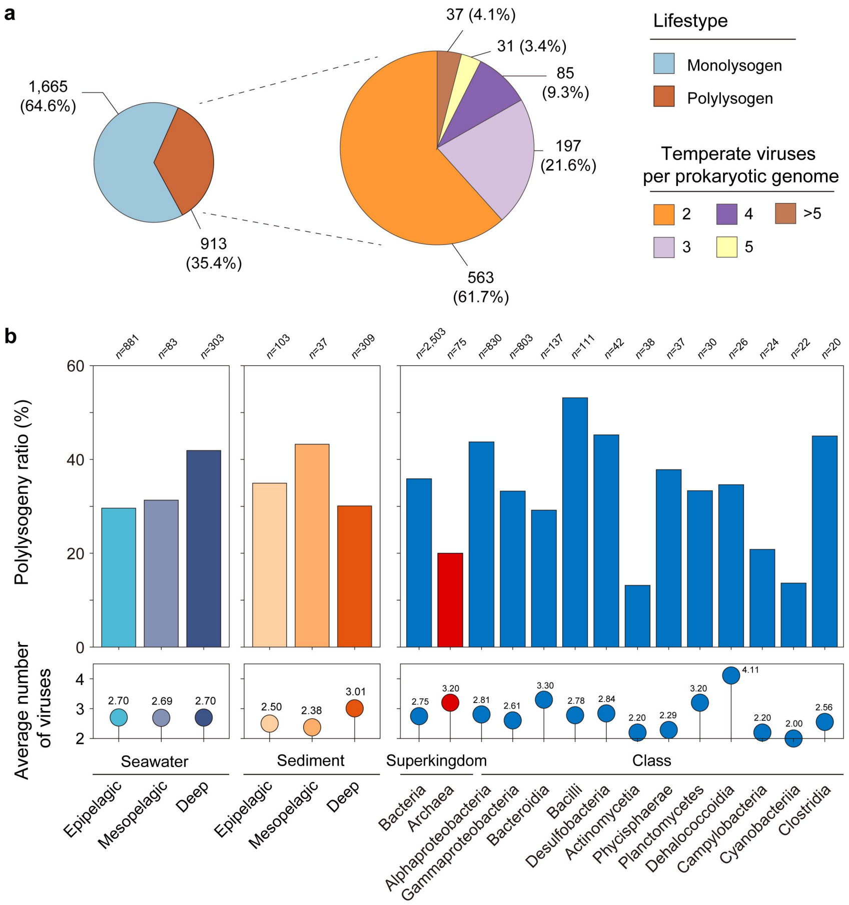

Fig. S9| Occurrence of polylysogeny in marine prokaryotes. a, Composition of marine polylysogens. The left pie chart indicates the percentage of polylysogenic genomes, and the right pie chart shows the percentages of the number of temperate viruses carried, considering only polylysogens. **b**, Rates of polylysogeny in different ocean zones (left two panels), prokaryotic superkingdom and classes (right panel). For clarity, only the classes with ≥ 20 genomes in which a temperate virus with a single copy marker protein was identified are displayed, and number of genomes contained in data groups are shown above bars. The average number of viruses of each group is shown above the filled circle.

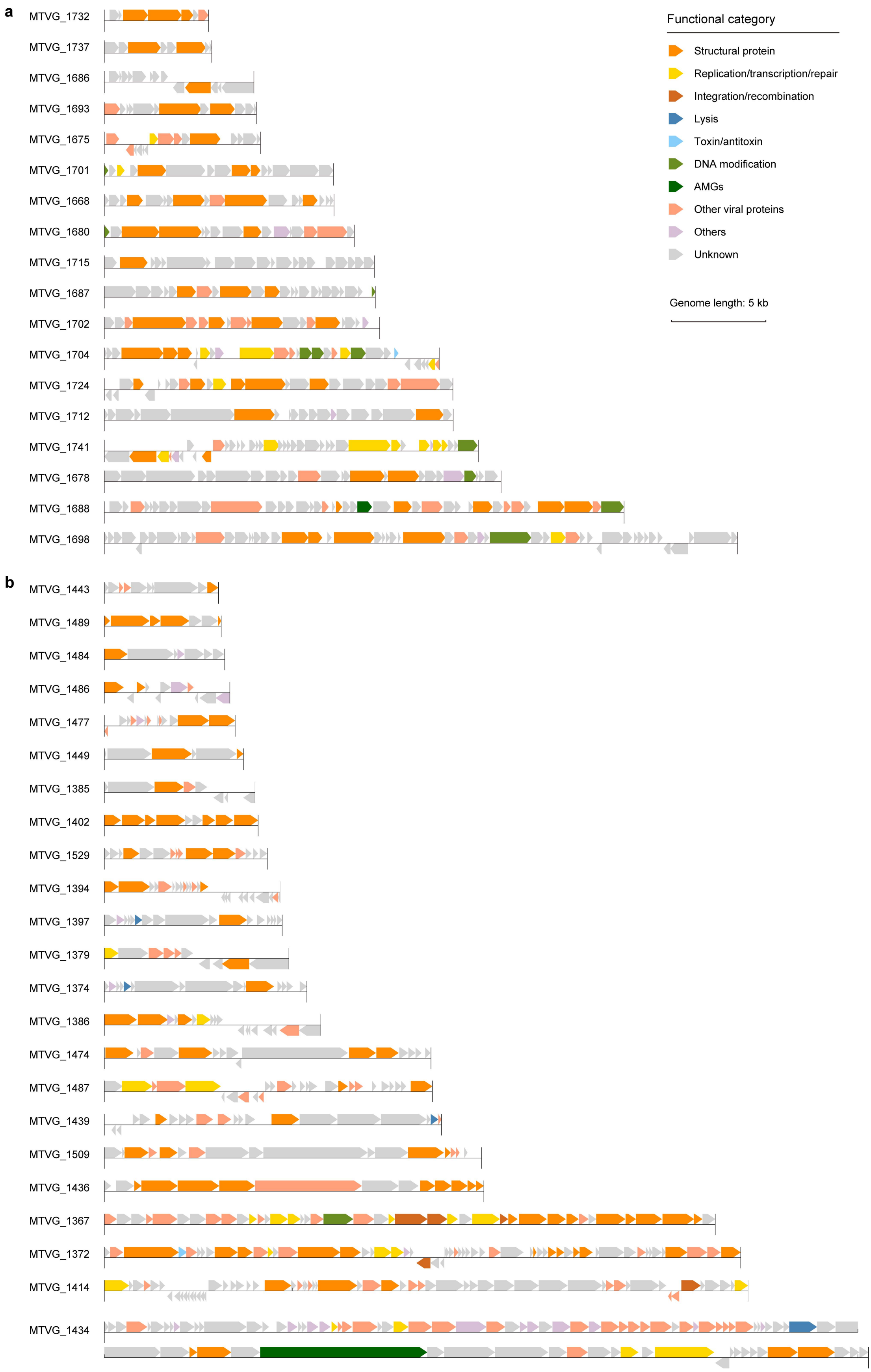

Fig. S10| Genome maps of temperate viruses in the representative marine

polylysogens *Hyphomonas* sp. IN11 (a) and *Dehalococcoidia* bacterium UBA6537 (b). The arrows depict the location and direction of predicted proteins on the viral genomes, and the fill colours indicate different functional categories of genes, as shown in the legend.

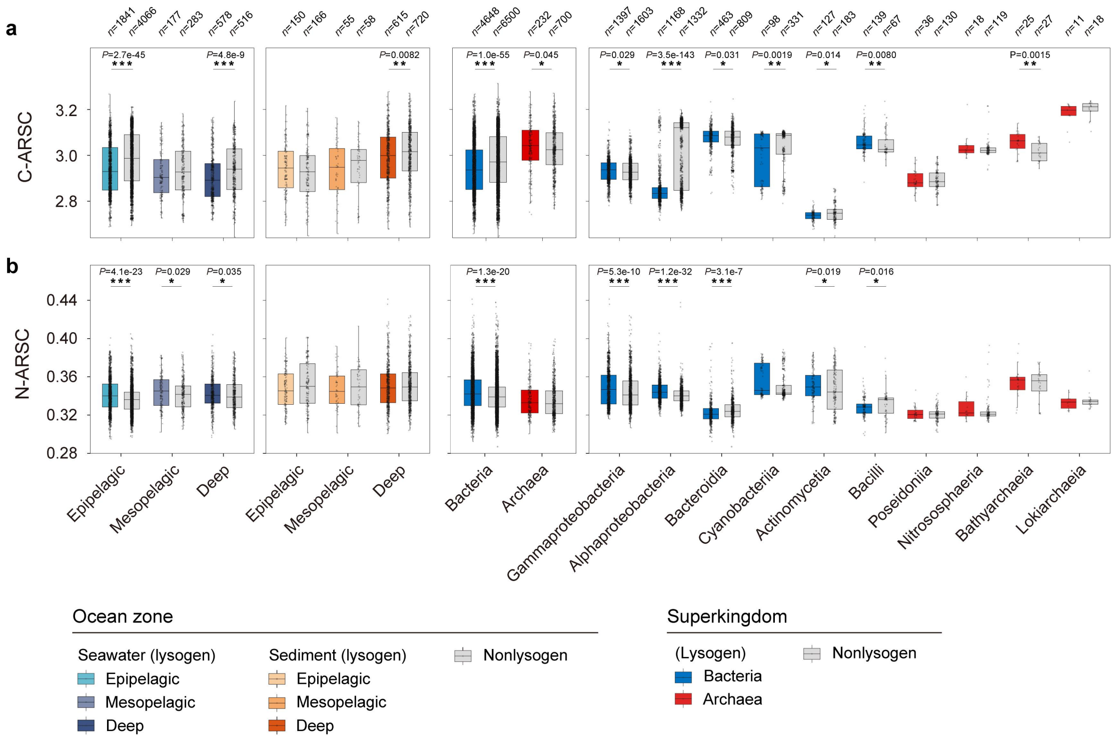

Fig. S11| Comparison of the carbon atoms per residue side chain (C-ARSC) (a) and nitrogen atoms per residue side chain (N-ARSC) (b) between marine lysogens and nonlysogens. Boxes representing lysogens are coloured according to the ocean zone (left two panels) or taxon (right two panels), and those representing nonlysogens are shown in light grey. All the significant differences between lysogens and nonlysogens and *P* values (two-sided Wilcoxon rank-sum test) are shown above the boxes. Each box represents the interquartile range (IQR), in which the middle line represents the median. The whiskers extend to 1.5×IQR, and all contained data are shown as the individual points. The number of genomes contained in each group (a and b) is shown at the top of the plot.

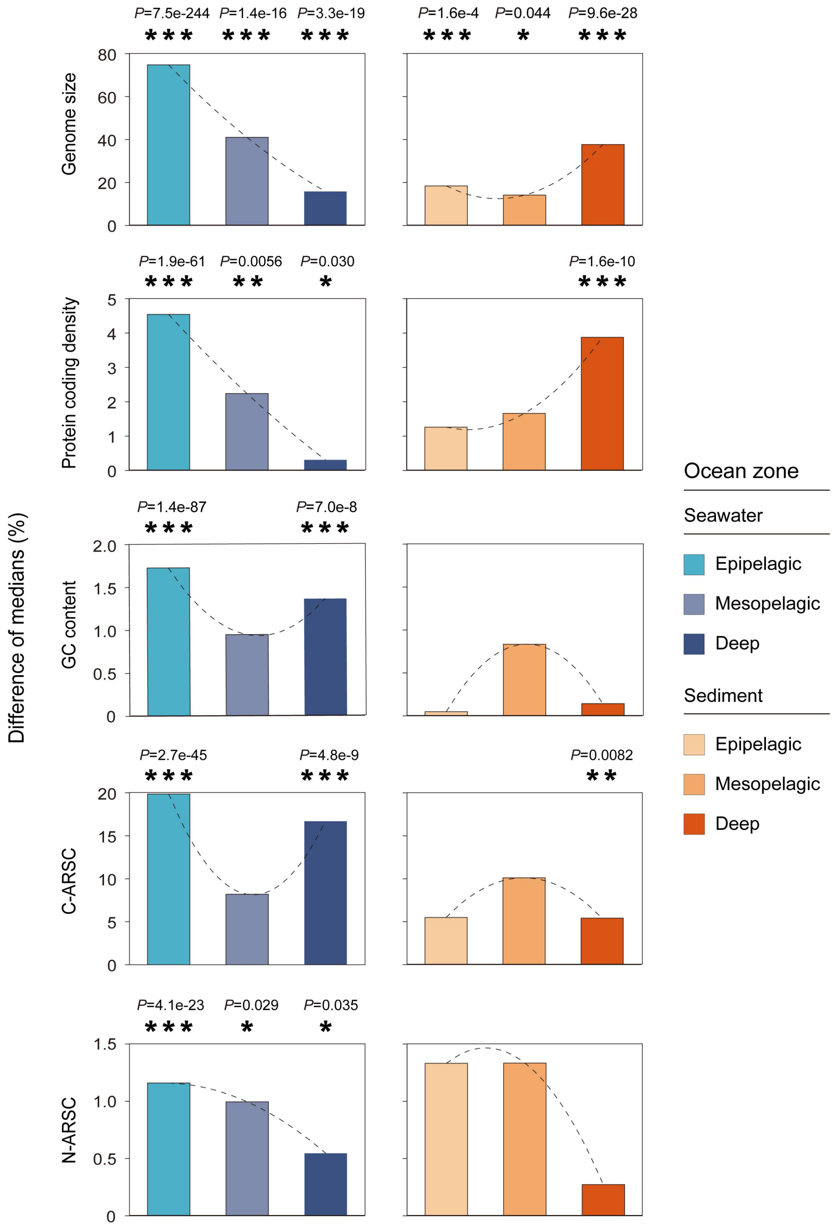

Fig. S12| Differences in genomic features between lysogens and nonlysogens in different ocean zones. Differences in medians were calculated as ($median_{high}$ - $median_{low}$)/$median_{low}$ and are shown as the bars. The dashed lines indicate the changes. All the significant differences between lysogens and nonlysogens were determined by two-sided Wilcoxon rank-sum tests, and the *P* values are shown above the bars. ****P* < 0.001; ***P* < 0.01; **P* < 0.05.

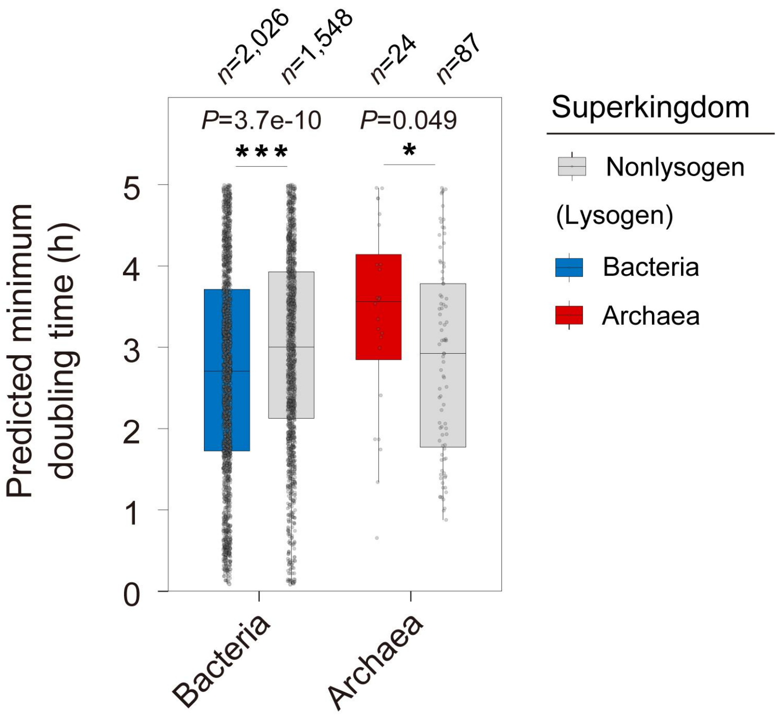

Fig. S13| Comparison of the predicted growth rates between marine lysogens and nonlysogens according to prokaryotic superkingdom. According to the instructions for practical application[4], only the genomes with an estimated minimal doubling time < 5 h were used in the analysis. Boxes representing lysogens are coloured according to superkingdom, and those representing nonlysogens are coloured light grey. All the significant differences between lysogens and nonlysogens and *P* values (two-sided Wilcoxon rank-sum test) are shown above the boxes. Each box represents the interquartile range (IQR), in which the middle line represents the median. The whiskers extend to 1.5×IQR, and all contained data are shown as points. The number of genomes contained in each group is shown at the top of the plot.

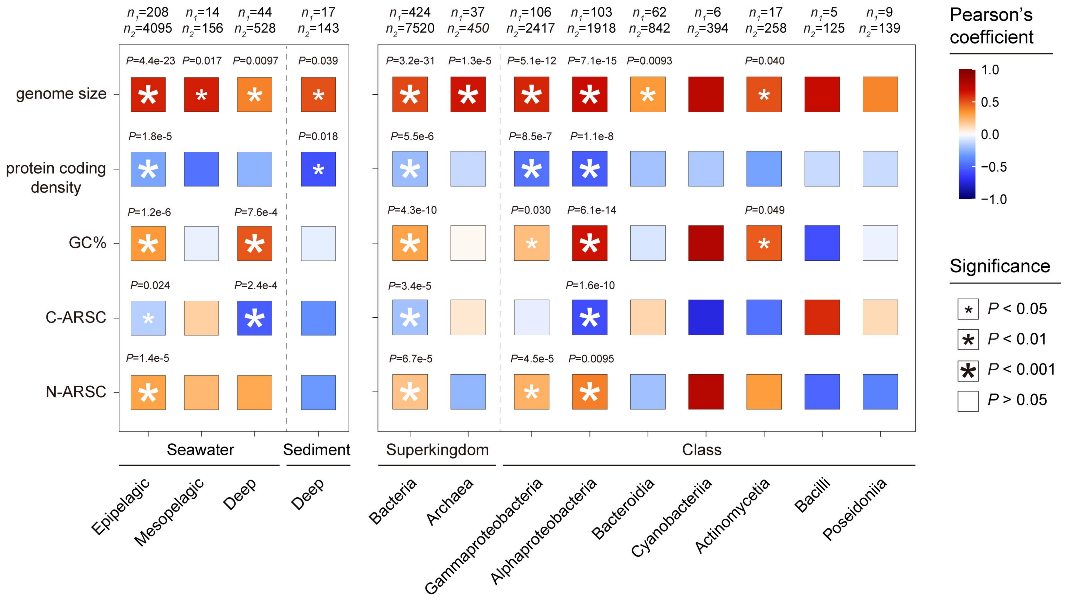

Fig. S14| Correlation analysis between lysogeny ratios (LyRs) and host genomic features. Pearson's correlation coefficients between the median genomic features and LyRs of genera (with ≥ 5 genomes) were calculated and are shown by the colour gradient of squares. The significant correlations were determined by two-sided tests and are marked by white asterisks, and the *P* values are represented by the sizes of the asterisks and shown above squares. The left and right panels display correlation data in different ocean zones and prokaryotic taxa, respectively. The number of genera used for the calculation of pearson's correlation (n_1) and genomes contained in the genera (n_2) in each group is shown at the top of the graphs.

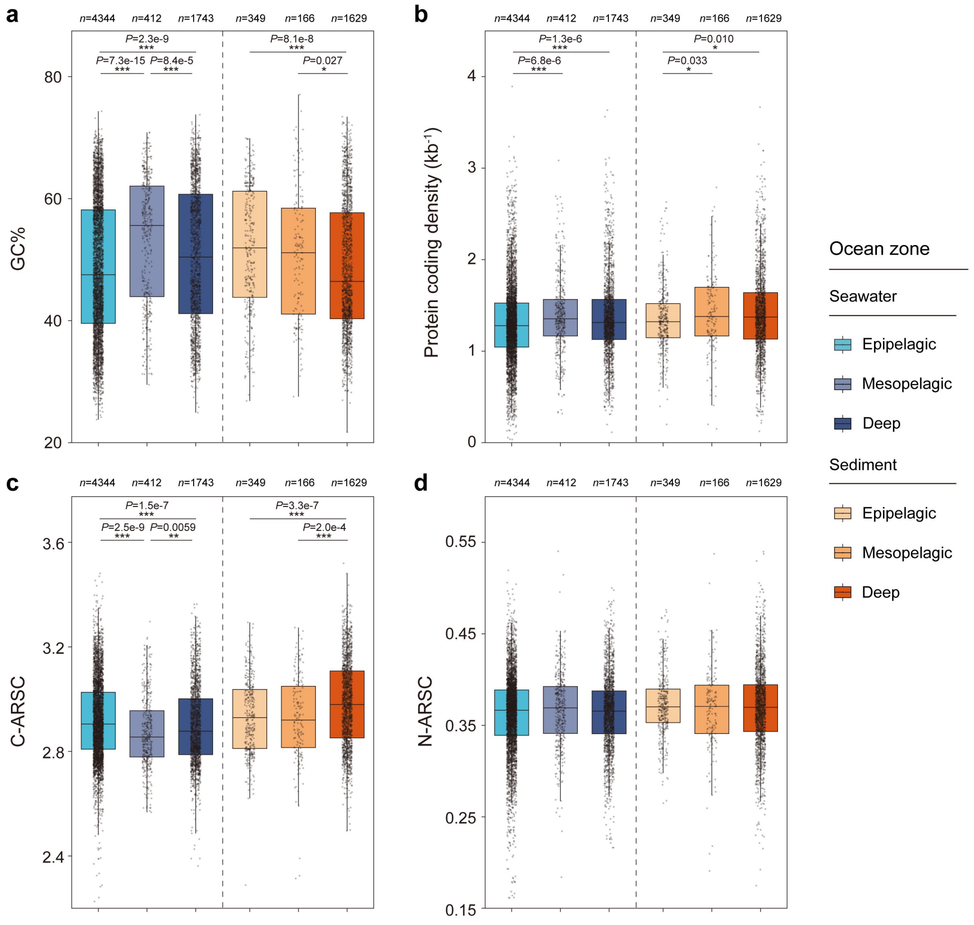

Fig. S15| Differentiation of temperate viruses in different ocean zones in terms of genomic features, including GC content (a), protein coding density (b), C-ARSC (c) and N-ARSC (d). The differences between different ocean zones were assessed by the two-sided Wilcoxon rank-sum test, and significant differences and *P* values are shown above boxes (other compared groups are shown in Supplementary Data 12). Each box represents the interquartile range (IQR), in which the middle line represents the median. The whiskers extend to 1.5×IQR, and all contained data are shown as points. The number of genomes contained in each group is shown above each box plot.

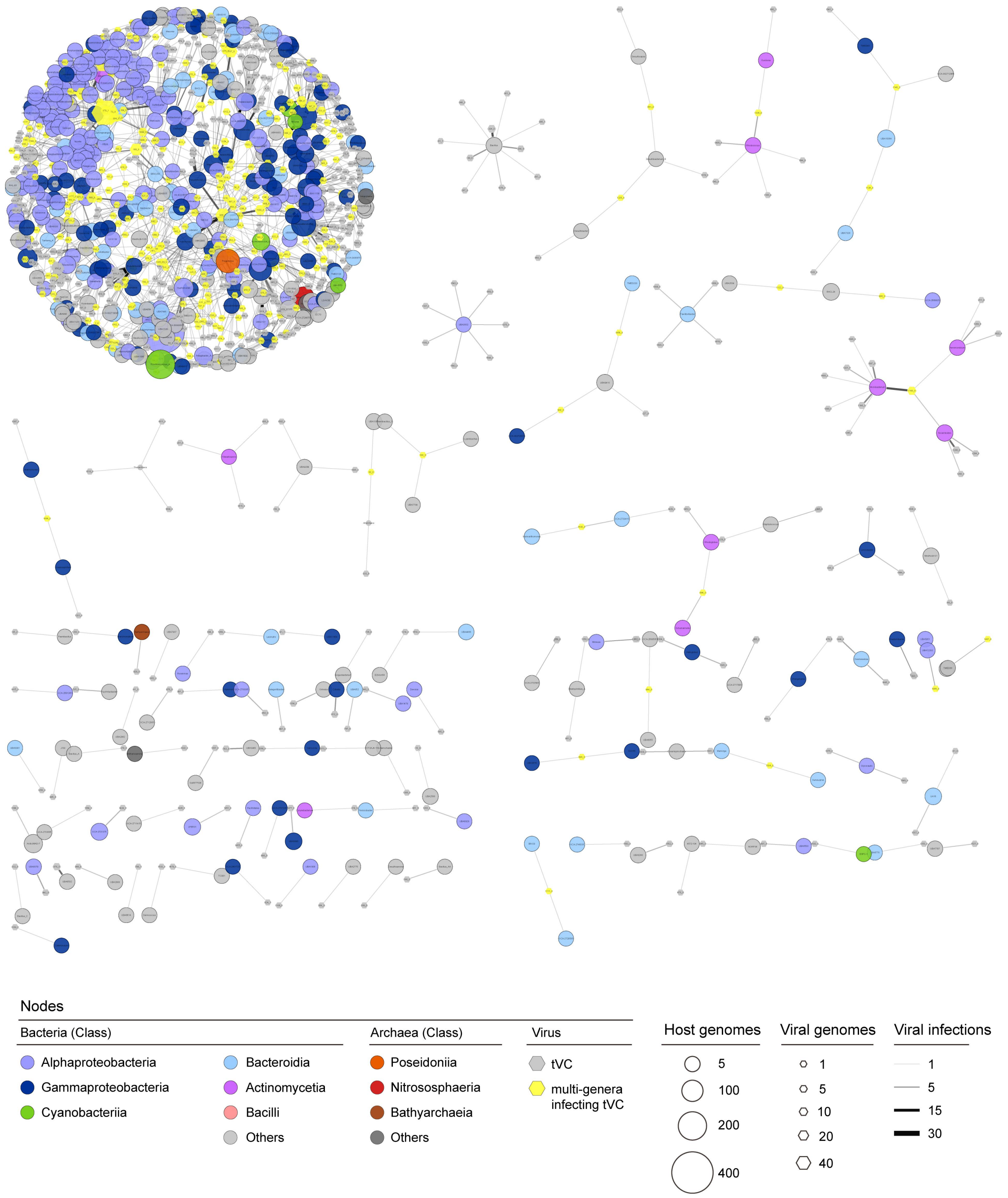

Fig. S16| Interaction network of prokaryotic genera and tVCs in seawater. The circles and hexagons represent host genera and tVCs, respectively, in which their names are shown. The sizes are proportional to the numbers of genomes included. The coloured circles represent different bacterial classes or archaeal phyla, and the yellow hexagons represent tVCs that infect multiple host genera. The numbers of infections are displayed as the shared edges and are proportional to the transparency and width. The network was visualized using the edge-weighted spring-embedded model, which places the host genera and tVCs with higher co-occurrence in closer proximity in the display.

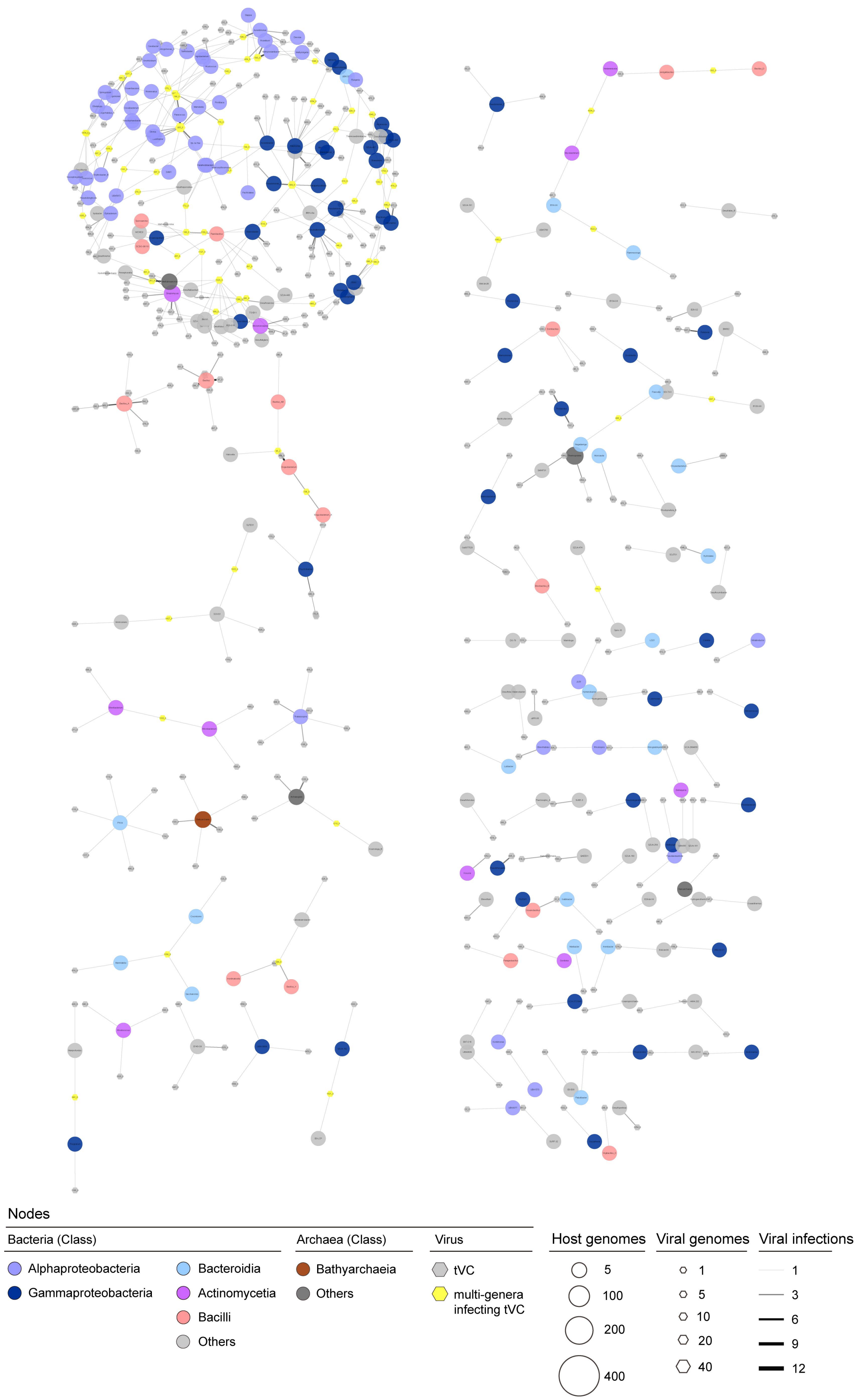

Nodes
Bacteria (Class)
Alphaproteobacteria
Gammaproteobacteria
Bacteroidia
Actinomycetia
Bacilli
Others
Archaea (Class)
Bathyarchaeia
Others
Virus
tVC
multi-genera infecting tVC
Host genomes
5
100
200
400
Viral genomes
1
5
10
20
40
Viral infections
1
3
6
9
12

Fig. S17| Interaction network of prokaryotic genera and tVCs in marine sediment. The circles and hexagons represent host genera and tVCs, respectively, in which their names are shown. The sizes are proportional to the numbers of genomes included. The coloured circles represent different bacterial classes or archaeal phyla, and the yellow hexagons represent tVCs that infect multiple host genera. The numbers of infections are displayed as the shared edges and are proportional to the transparency and width. The network was visualized using the edge-weighted spring embedded model, which places the host genera and tVCs with higher co-occurrence in closer proximity in the display.

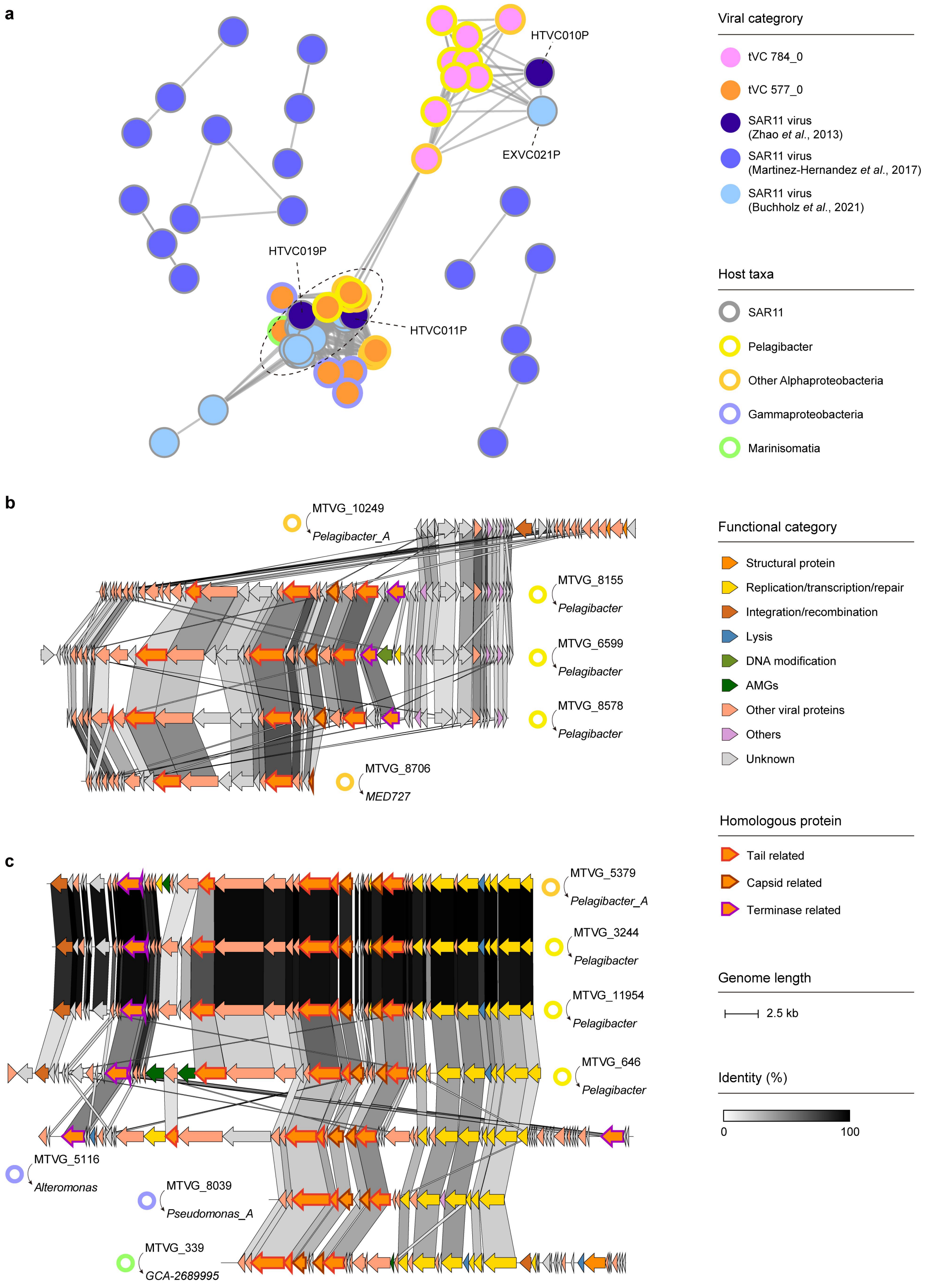

Fig. S18| Overview of temperate viral clusters infecting SAR11. a, Protein sharing network of SAR11 viruses. The pink and orange nodes represent temperate viruses in tVC 784_0 and 577_0 that were identified in this study, and the other nodes represent SAR11 viruses reported previously. The coloured borders show host taxa, and the shared edges

indicate shared protein content. The network was visualized using the edge-weighted spring-embedded model, which places the viruses sharing higher protein content in closer proximity in the display. **b**, **c**, Genome maps of temperate viruses in tVC 784_0 (**b**) and 577_0 (**c**). The gene comparison of viruses was performed and visualized by clinker (v0.0.27)[5]. The homologous regions between adjacent genomes are indicated by the shared areas. The bar for percent protein identity is outlined on the right. The viral ID and host genus/class of each virus are shown next to each genome map. The arrows depict the location and direction of predicted proteins on the viral genomes. The fill and outline colours indicate different functional categories of genes and specific homologous structural proteins, respectively, as shown in the legend.

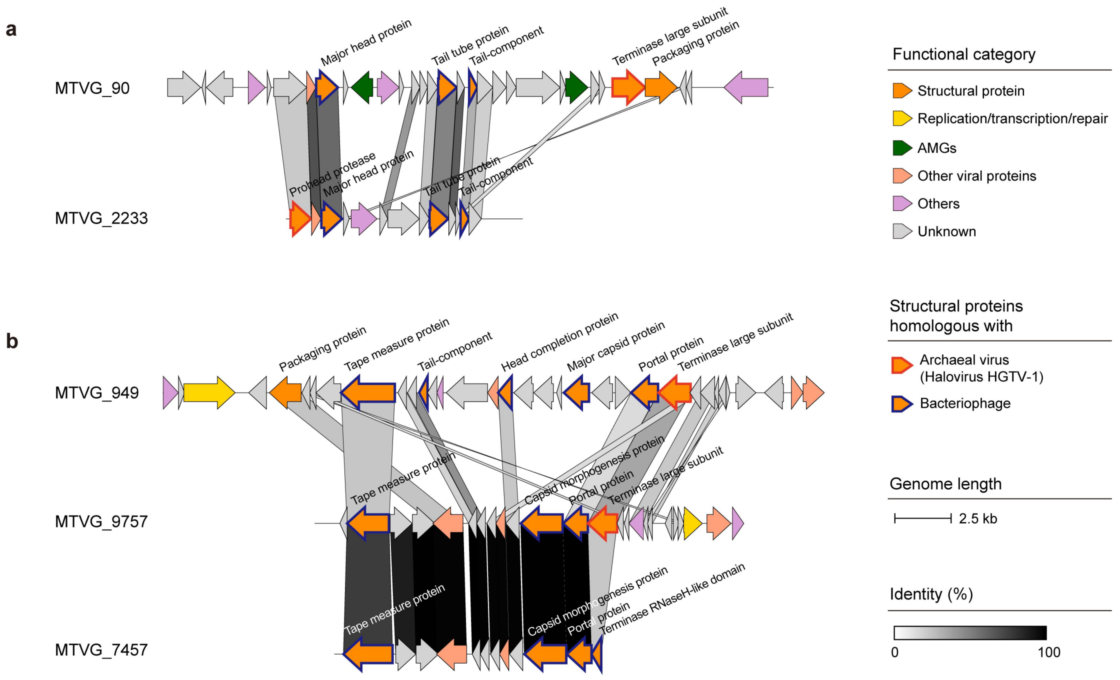

Fig. S19| Genome maps of representative tVCs 1190_0 (a) and 1191_0 (b) that infect Bathyarchaeia. The gene comparison of viruses was performed and visualized by clinker (v0.0.27)[5]. The homologous regions between adjacent genomes are indicated by the shared areas. The bar for percent protein identity is outlined on the right. The viral ID is shown next to each genome map. The arrows depict the location and direction of predicted proteins on the viral genomes. The specific homologous structural proteins are marked upon corresponding arrows. The fill and outline colours indicate different functional categories of genes and structural proteins homologous with bacteriophages or archaeal viruses (Halovirus HGTV-1), respectively, as shown in the legend.

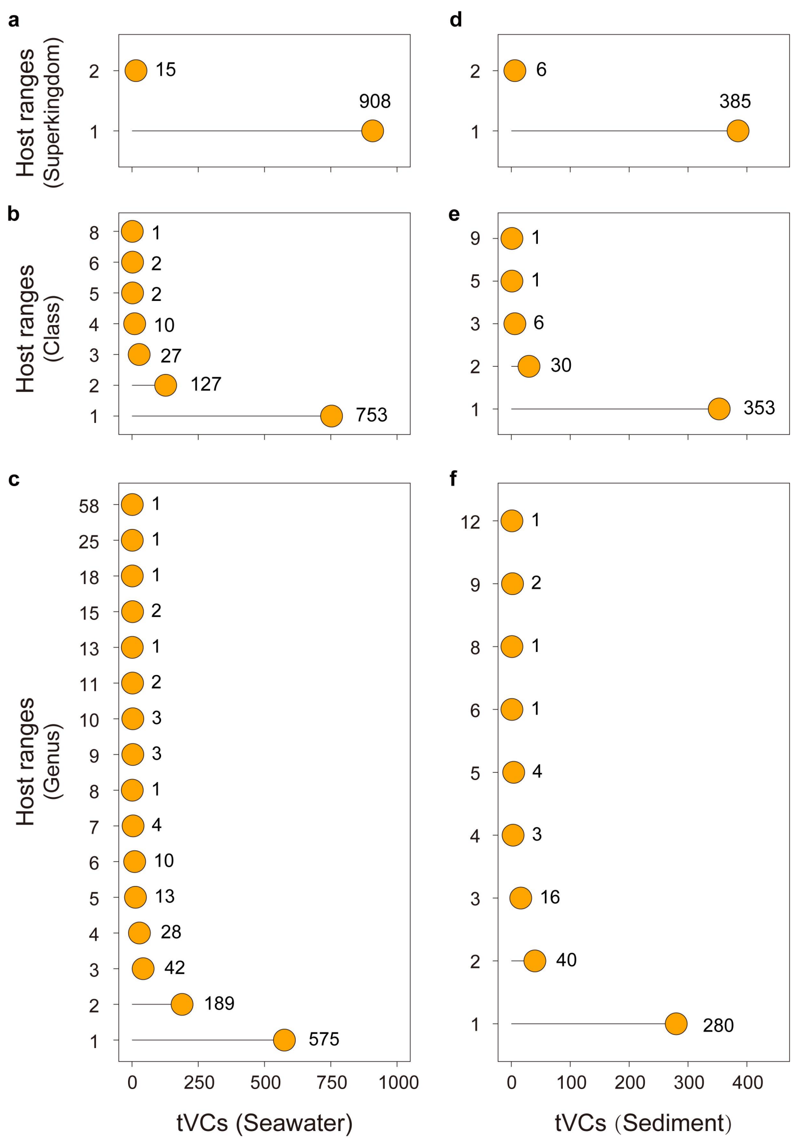

Fig. S20| Host ranges of marine temperate viral clusters in seawater (a-c) and sediment (d-f). The number of host superkingdom (**a, d**), class (**b, e**) and genus (**c, f**) infected by marine tVCs are displayed to the right of the filled circles.

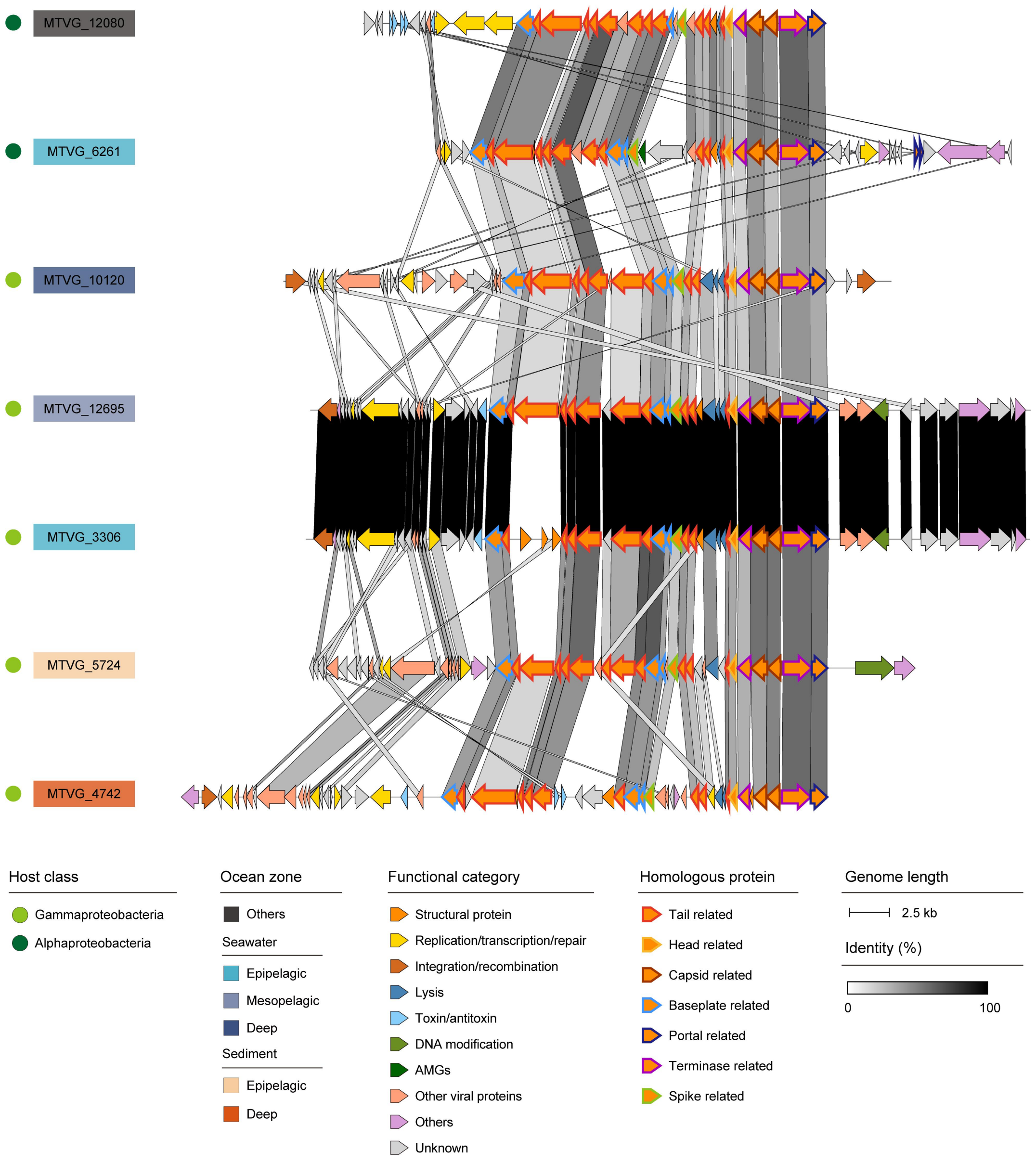

Fig. S21| Genome maps of representative temperate viruses in tVC 409_0. Viruses with the largest genome size in tVC 409_0 that infect Gammaproteobacteria in different ocean zones as well as two viruses in this viral cluster that infect Alphaproteobacteria are displayed. The gene comparison of viruses was performed and visualized by clinker (v0.0.27)[5]. The homologous regions between adjacent genomes are indicated by the shared areas. The bar for percent protein identity is outlined on the bottom right. The derived ocean zone and host class of each virus are shown as the coloured rectangle and circle, respectively. The arrows depict the location and direction of predicted proteins on the viral genomes. The fill and outline colours indicate different functional categories of

genes and specific homologous structural proteins, respectively, as shown in the legend.

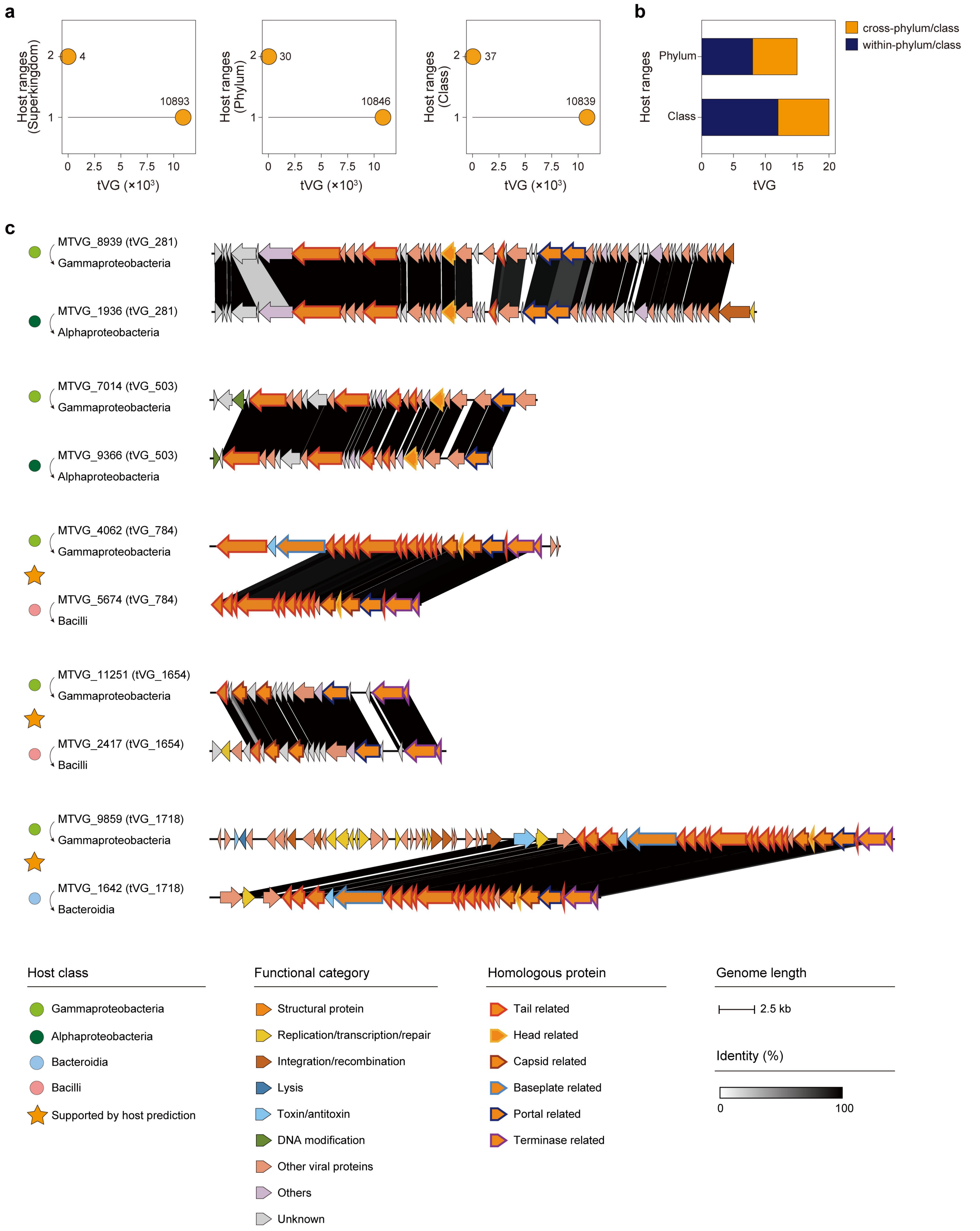

Fig. S22| Host ranges of marine temperate viral genera (tVG) (a), host prediction of multiphylum- and multiclass-infecting tVG (b) and genome maps of representative broad-host-range tVG (c). a, The number of host superkingdom, phylum and class infected by marine tVG are displayed next to the filled circles. **b**, Verification of cross-phylum/class infecting tVG by host prediction based on homology of CRISPR spacers and nucleotide sequences. The 4 multisuperkingdom-infecting, 30

multiphylum-infecting and 37 multiclass-infecting tVG were analyzed, and the 4 tVG showing cross-superkingdom host range were not supported by host prediction against the MPGD and RefSeq prokaryotic genomes, therefore not shown in the diagram. **c**, Viruses in representative tVG with the largest genome size that infect each host class are displayed. The gene comparison of viruses was performed and visualized by clinker (v0.0.27)[5]. The homologous regions between genomes are indicated by the shared areas. The bar for percent protein identity is outlined on the bottom right. The viral ID and host class (indicated by a coloured circle) of each virus are shown at the left of each genome map. The arrows depict the location and direction of predicted proteins on the viral genomes. The fill colours indicate different functional categories of genes, and the outline colours indicate specific homologous structural proteins, as shown in the legend.

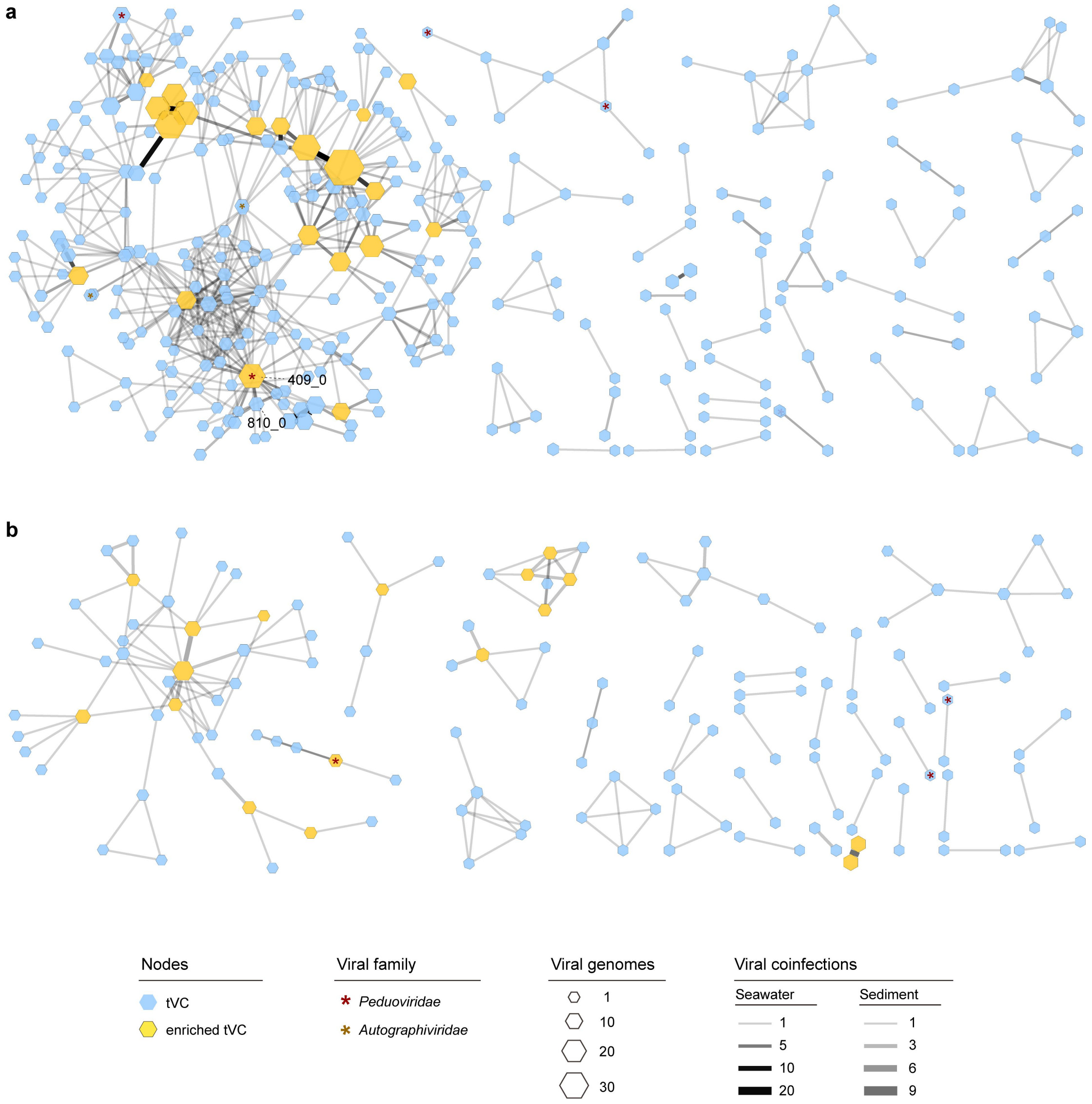

Fig. S23| Coinfection networks between tVCs in seawater (a) and sediment (b). The nodes represent tVCs, and the sizes are proportional to the number of contained genomes. The shared edges represent the number of temperate viral coinfections. The tVCs with ≥ 15 genomes in seawater or ≥ 5 genomes in sediment are shown in light orange. The coloured asterisks show the taxonomy of the assigned viral family. The networks were visualized using the edge-weighted spring-embedded model, which places the tVCs with a higher number of coinfections in closer proximity in the display. To avoid overestimating the number of coinfections, only the tVCs with single-copy marker proteins were included in the networks.

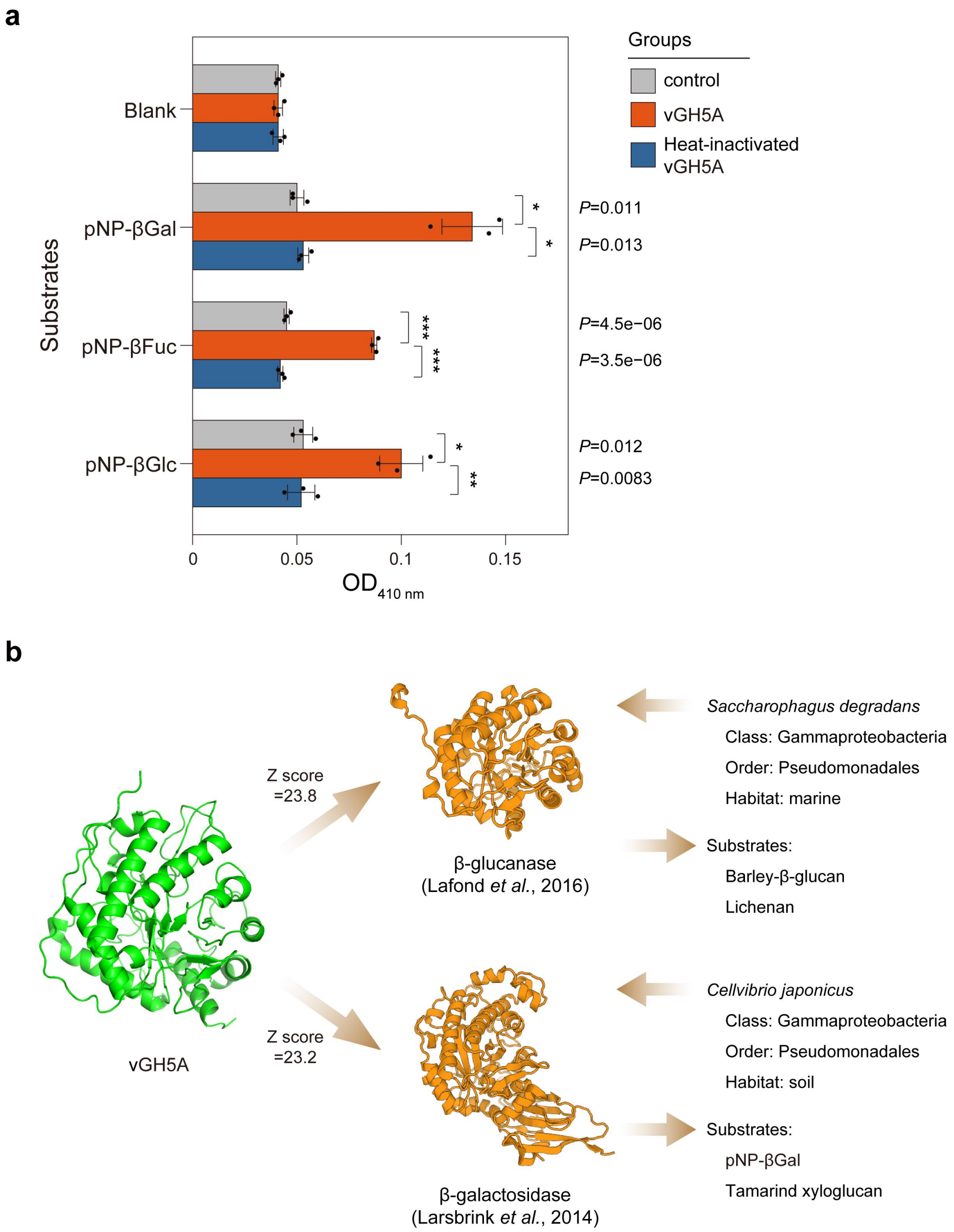

Fig. S24| Functional characterization of the viral glycoside hydrolase vGH5A. a, Enzymatic activity assays of vGH5A. Enzyme activity was shown by the increase in the absorbance at 410 nm upon addition of vGH5A. For all the enzymatic assays, the reaction mixtures without enzyme and with the heat-inactivated enzyme were used as the blank and the negative control, respectively. The data from one of three independent experiments are shown, and the error bars indicate the standard deviation, which was based on three technical replicates. All significant differences between groups were determined by two-sided unpaired Student's *t* tests, and the *P* values are shown at the

right of the bars. **b**, Structural comparison between vGH5A and representative microbial glycoside hydrolases with experimentally verified functions and structures. The 3D structure of vGH5A was predicted by Alphafold[6] and visualized by PyMOL[7]. The structural similarities were calculated using DALI[8], and the Z scores are indicated. 3D structures of experimentally verified β-glucanase (PDB ID: 5A94) and β-galactosidase (PDB ID: 4D1J) were downloaded from DALI. Source bacteria (along with related information) and substrates of the reference enzymes are marked.

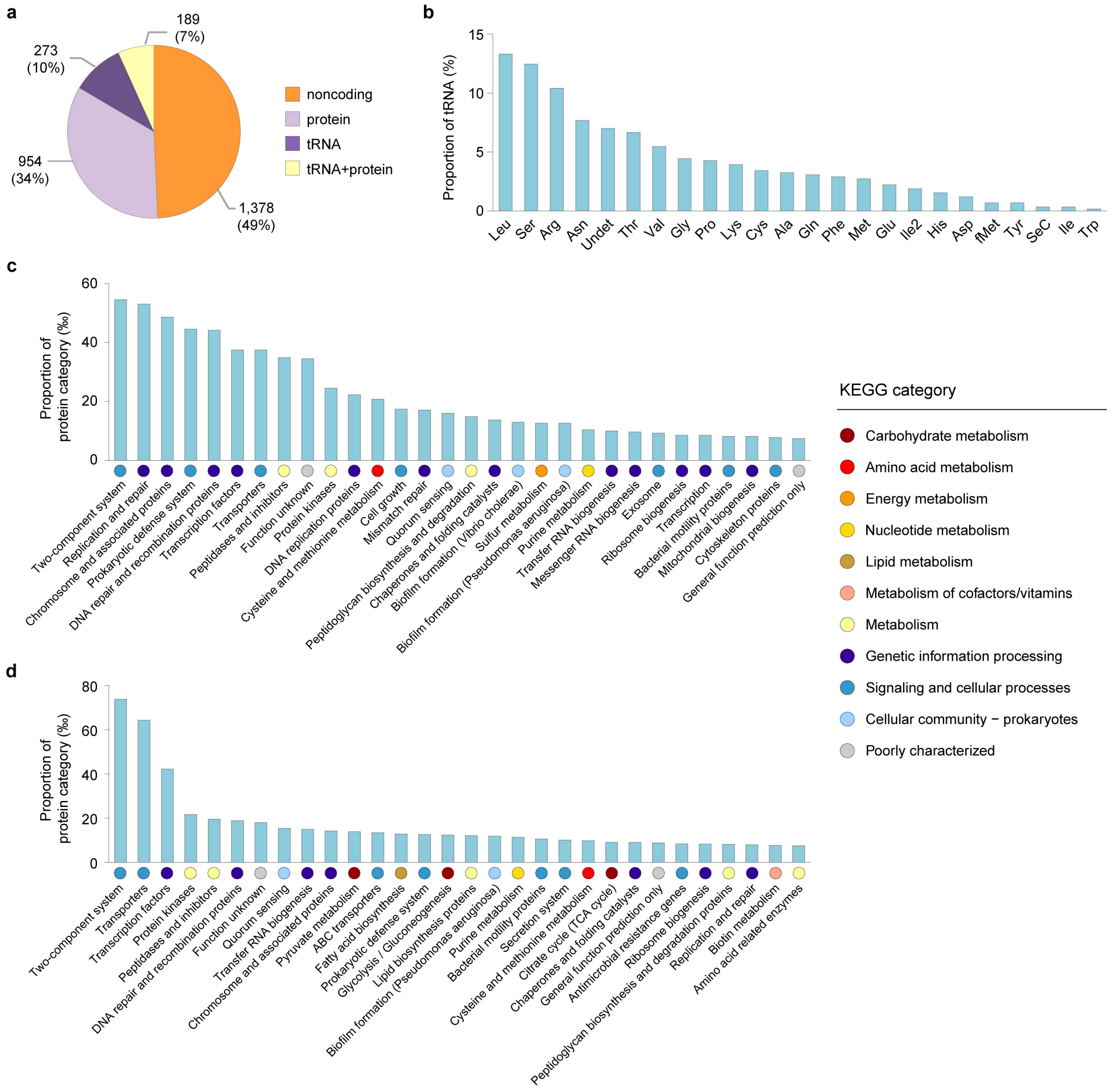

Fig. S25| Overview of integration sites of marine temperate viruses. a, Total composition of integration sites. **b**, Composition of coded tRNAs at integration sites. **c**, Composition of coded proteins at integration sequences. **d**, Composition of the genomic context of integration sites. The 5 host proteins flanking the integrated viral genome (upstream and/or downstream) were analysed. The proteins (**c**, **d**) were annotated via online BLASTp alignment against the KEGG database using default parameters, and functional categories are indicated by the coloured circles.

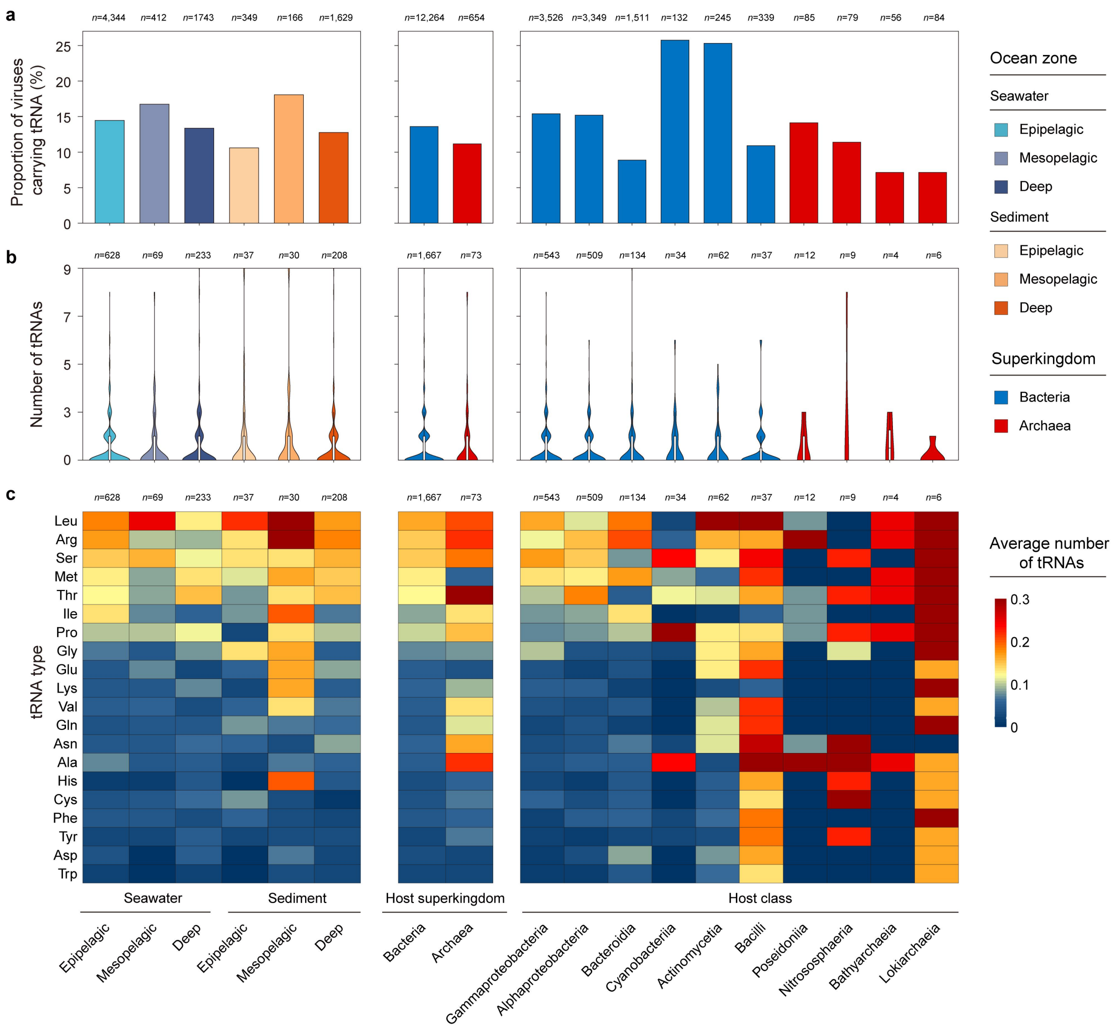

Fig. S26| Overview of tRNAs encoded by marine temperate viruses. a, Percentage of tRNAs-carrying marine temperate viruses. **b**, Numbers of tRNAs encoded by marine temperate viruses. The violin plots show the data distribution based on kernel density estimation. Each inner box represents the interquartile range (IQR), in which the middle line represents the median. The whiskers extend to 1.5×IQR. The differences between different ocean zones and superkingdom were assessed by the two-sided Wilcoxon rank-sum test; no pair of comparisons between ocean zones and prokaryotic superkingdom showed a significant difference. **c**, The composition of tRNA types encoded by marine temperate viruses. The data are grouped and analysed among different ocean zones (left panel) and host taxa (right two panels). The number of genomes contained in each group is shown upon each graph.

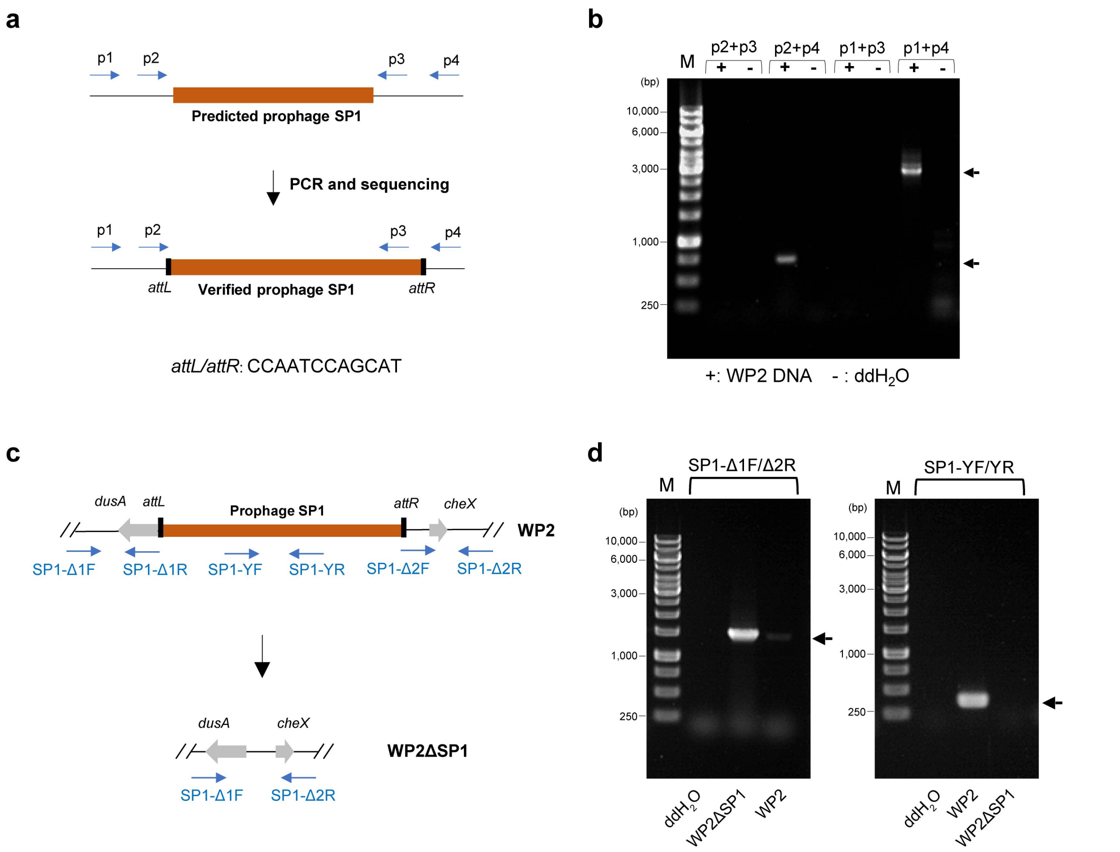

Fig. S27| Determination of the SP1 boundary in the WP2 genome (a, b) and construction of the SP1 deletion mutant (c, d). a, Schematic of the determination of the exact start and end sites in the WP2 genome by PCR and sequencing. The locations of the primer pairs used for PCR amplification are shown with blue arrows. **b**, The electrophoresis of PCR products. The primer pairs and template DNA used for PCR are indicated for each lane, and the target bands are marked with dark arrows. M, DNA size marker. **c**, Schematic of the construction of the SP1 deletion mutant WP2ΔSP1. The locations of the primer pairs used for mutant construction and verification are shown with blue arrows. **d**, Verification of SP1 deletion by PCR. The primer pairs and template DNA used for PCR amplification are indicated for each lane, and the target bands are marked with dark arrows. M, DNA size marker. The uncropped scans of gels in panel **b** and **d** are provided at the end of the supplementary Information file.

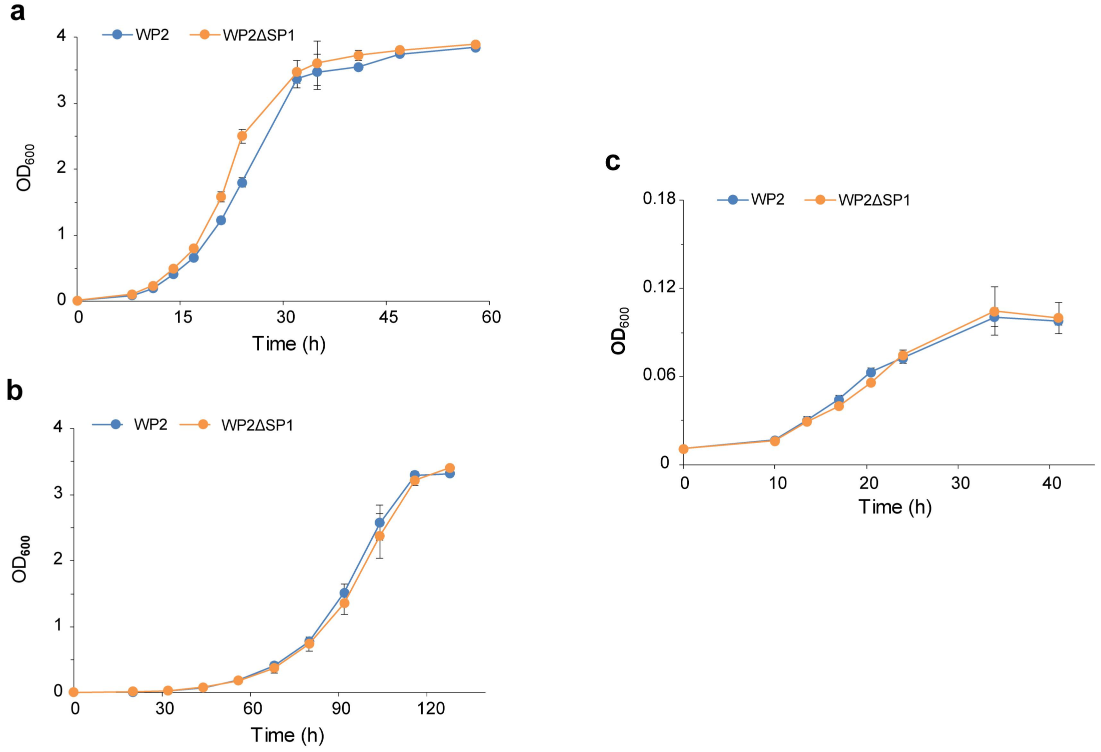

Fig. S28| The growth curve of WP2ΔSP1 under different cultivation conditions. a, 0.1 MPa and 15 °C; **b**, 0.1 MPa and 4 °C; **c**, 20 MPa and 4 °C. The growth of the strains in 2216E medium was detected at OD_{600nm}. Data are represented as the mean ± SD and are based on three biologically independent samples.

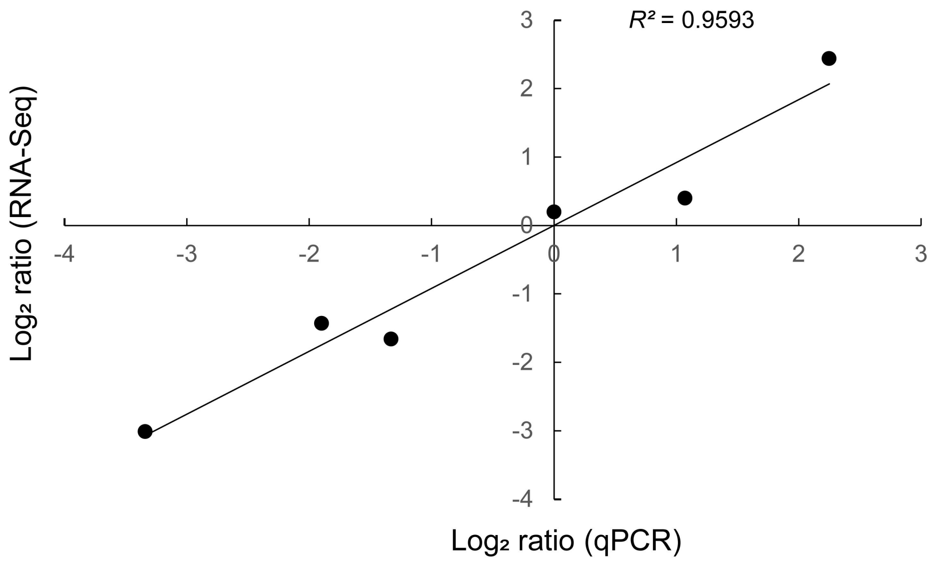

Fig. S29| Correlation analysis of the RNA-seq and RT-qPCR assays. Six genes with different expression levels were selected randomly for this assay. The RT-qPCR log_2 values were plotted against the RNA-seq log_2 values, and the correlation coefficient (R^2) is shown on the plot.

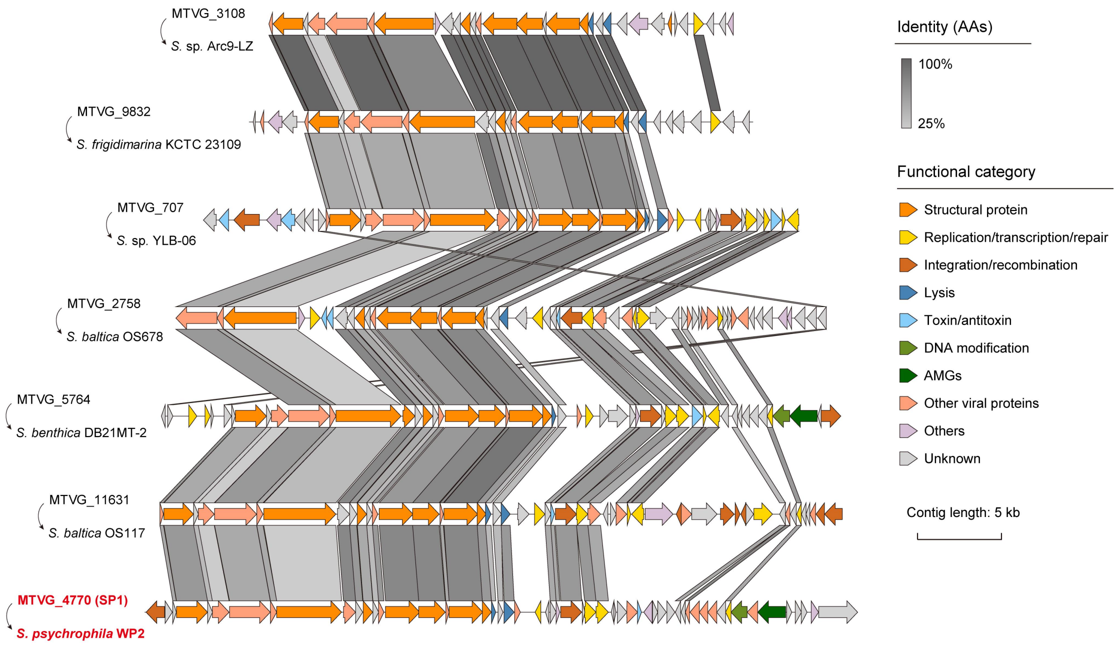

Fig. S30| Genomic maps depicting SP1-like prophages in marine *Shewanella* species. The arrows depict the location and direction of predicted proteins on the phage genomes, and the fill colours indicate different gene functional categories, as shown in the legend. The protein comparison of SP1 and other prophages was performed by BLASTp alignment with an e-value cut-off of 1e-5 and visualized by Easyfig[5]. The homologous regions between adjacent genomes are indicated by the shared areas. The bar for percent protein identity is outlined on the right.

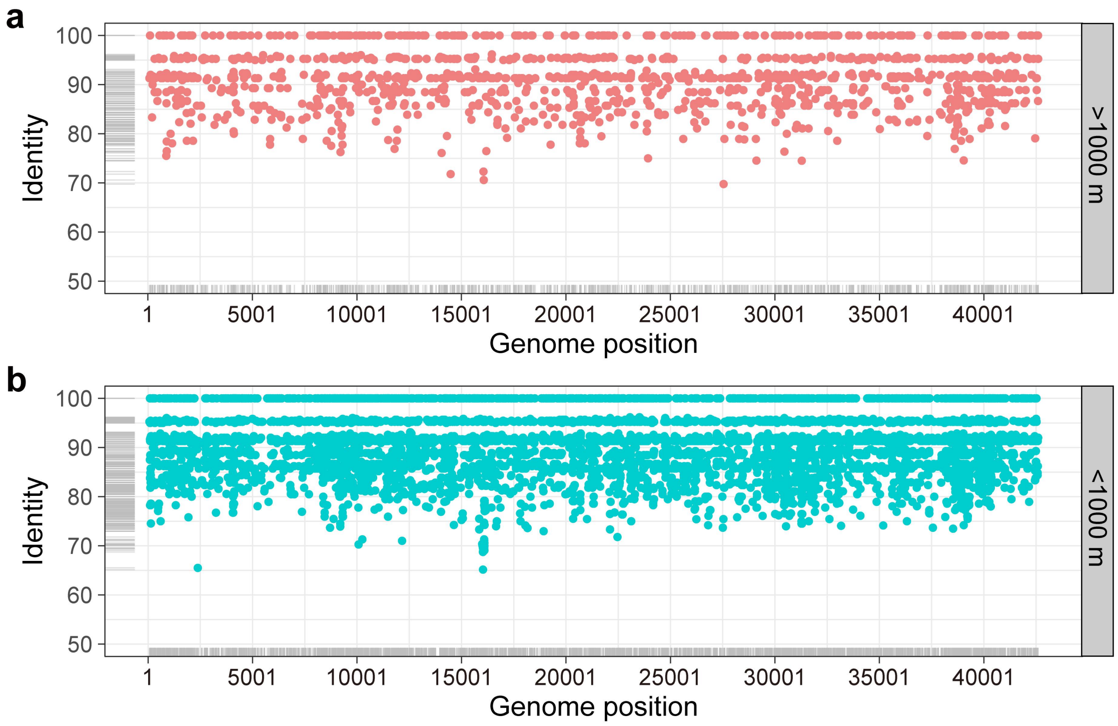

Fig. S31| Recruitment analysis of SP1-like viruses in the marine virome. Recruitment plots were computed using metagenomic raw reads from the POV with water depths <1,000 m (**a**) and ≥1,000 m (**b**). The recruitment analyses are performed by BLASTn with an e-value cut-off of ≤ 10^{-3}. Only reads that hit with ≥50% identity are shown.

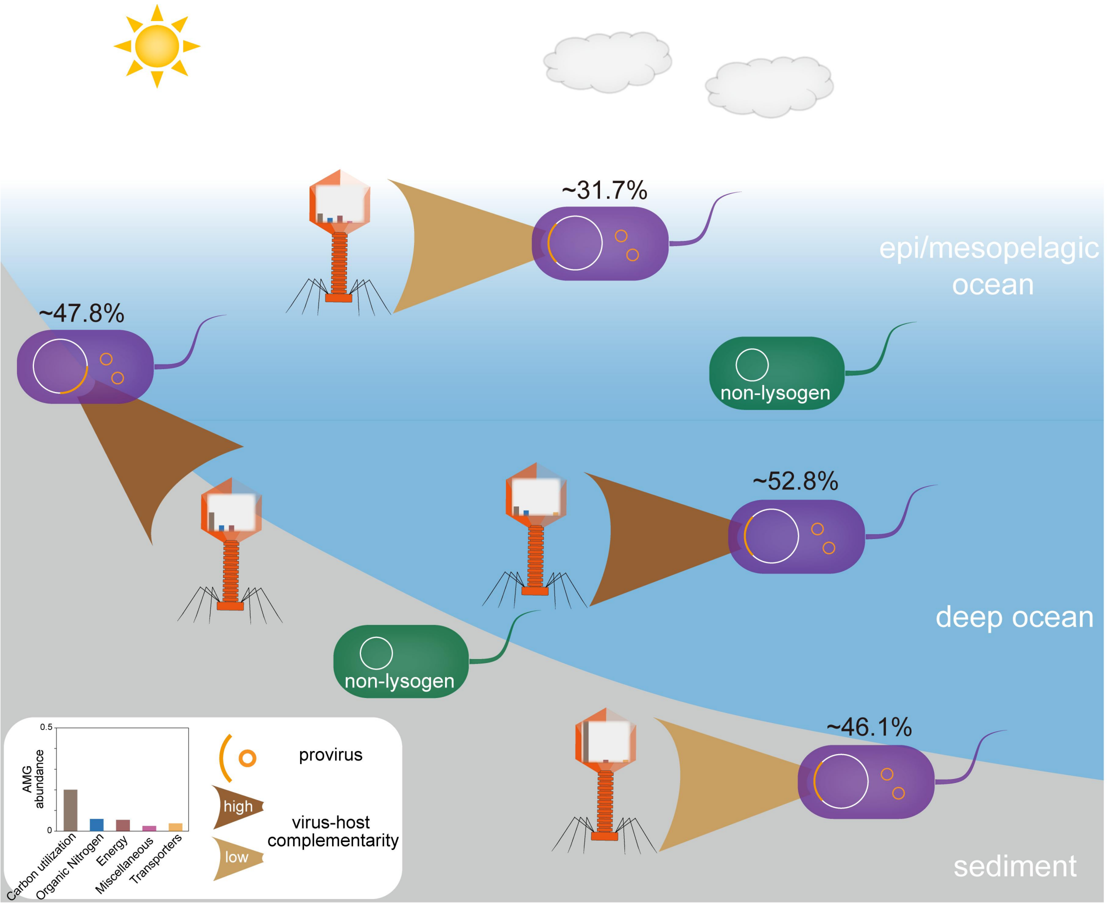

Fig. S32| Schematic depiction of lysogens and temperate viruses in the ocean. The main findings in the present study are shown in a simplified manner. Purple and green cells represent lysogens and nonlysogens, respectively; lysogens generally possess a larger genome size. The histogram in the virion head shows the relative abundance of different functional AMGs carried by temperate viruses in different marine environments. The colour shades of the block connecting the virus and the host represent the general degree of virus-host complementarity. To enhance understanding, the figure was not drawn to scale in terms of the ocean zones, and the epipelagic and mesopelagic zones were combined to facilitate comparison with the deep ocean.

Table S1. Bacterial strains and plasmids used in this study.

Strain or plasmid	Relevant genotype	Reference or source
E. coli strains		
WM3064	Donor *E. coli* strain for conjugation; Δ*dapA*	Lab stock
BL21 (DE3)	Recombinant protein expression host	GE Healthcare
Rosseta (DE3)	Recombinant protein expression host	Novagen
S. psychrophila WP2 Strains		
WP2	*S. psychrophila* WP2 wild-type strain	[7,8]
WP2ΔSP1	Wild-type WP3 strain with prophage SP1 deletion	This work
Plasmids		
pRE112	Allelic-exchange vector; Chl^r, *sacB*	[9]
pRE112-SP1	pRE112 containing the PCR fragment for deleting SP1 prophage	This work
pET28a	His-tag protein expression vector; Kan^r	Novagen
pMAL-c2x	MBP-tag protein expression vector; Amp^r	New England Biolabs

Table S2. Primers and oligonucleotides used in this study.

Name	Sequence (5'-3')	Description
T7	TAATACGACTCACTATAGGG	Plasmid verification
T7 Ter	TGCTAGTTATTGCTCAGCGG	Plasmid verification
pMal-F	GGTCGTCAGACTGTCGATGAAGCC	Plasmid verification
pMal-R	TGTAAAACGACGGCCAGT	Plasmid verification
pMal-Agar-F	ATTTCAGAATTCGGATCCATGGATATTACCGAAAAATTATTCCC	Vector construction
pMal-Agar-R	CAGTGCCAAGCTTGCTTAATGATGATGATGATGATGCAAATTAATAATAC	Vector construction
pMal-line-F	GGATCCGAATTCTGAAAT	Vector construction
pMal-line-R	GCAAGCTTGGCACTG	Vector construction
SP1-Δ1F	cgatcccaagcttcttctagaGCAGAGTAGTTGATCGACCTCAGC	SP1 deletion
SP1-Δ1R	attgcgcctATGCTGGATTGGACGGATCG	SP1 deletion
SP1-Δ2F	caatccagcatAGGCGCAATTGAGAAGGTGC	SP1 deletion
SP1-Δ2R	catgaattcccgggagagctcGGTGAATGGCATGATGATCTTCA	SP1 deletion
ChlFor	TAAATACCTGTGACGGAAGAT	Deletion verification
ChlRev	TATCACTTATTCAGGCGTAGC	Deletion verification
SP1-p1	CTCCCAGAAAGCGTCACCAT	Boundary determination
SP1-p2	TGACGCACTCAGCTACCAAT	Boundary determination
SP1-p3	TCGCAATGGCATCCTTTGGT	Boundary determination
SP1-p4	TGGTTTTCCAGGGCTGAGTT	Boundary determination
SP1-p5	AGAGCGTTCCAGTTCTGCTA	Excision Verification
SP1-p6	TACTGAACCAGCAGTTGGCG	Excision Verification

SP1-YF	ACTCAAAGAGAGGTGACCACA	SP1 verification
SP1-YR	CTGTGCGTAGGCGTTAGTCT	SP1 verification
sps_RS18235-RT-F	GGCGAAGCGCCTGATG	qPCR
sps_RS18235-RT-R	GCTCGCTTAGATGCCAGGAA	qPCR
sps_RS27395-RT-F	CGTGATCTCGAGTGGCATCA	qPCR
sps_RS27395-RT-R	ACAAGCGCAGGGCCTTAA	qPCR
sps_RS03395-RT-F	GCCATGGCGGTGAATGAA	qPCR
sps_RS03395-RT-R	TTGGTGGGCGCGGTAA	qPCR
sps_RS13515-RT-F	GCGCCGCATAGGTTTCC	qPCR
sps_RS13515-RT-R	CGAAGTCAGAGTGCGTTGCA	qPCR
sps_RS22770-RT-F	TGGGCCGATACCGTTGACT	qPCR
sps_RS22770-RT-R	CCCATCTGCCTATTTCGATACAC	qPCR
sps_RS25990-RT-F	ACCCGACGCTCATTTGTGA	qPCR
sps_RS25990-RT-R	GGAACATCCCTCTAAGTGGGTTAA	qPCR
rho-RT-F	CGGCCCGGATGACATCTAT	qPCR
rho-RT-R	CCGGTTCGCATGCTGAAG	qPCR
SP1-RT-F	GCAGACATTATTCGAGCTAG	qPCR
SP1-RT-R	GTCAATCATGTCATCATCCC	qPCR

Supplementary References

1 Parks, D. H., Imelfort, M., Skennerton, C. T., Hugenholtz, P. & Tyson, G. W. CheckM: assessing the quality of microbial genomes recovered from isolates, single cells, and metagenomes. *Genome Res.* **25**, 1043-1055, doi:10.1101/gr.186072.114 (2015).

2 Nayfach, S. *et al.* CheckV assesses the quality and completeness of metagenome-assembled viral genomes. *Nat. Biotechnol.* **39**, 578-585, doi:10.1038/s41587-020-00774-7 (2021).

3 Weissman, J. L., Hou, S. & Fuhrman, J. A. Estimating maximal microbial growth rates from cultures, metagenomes, and single cells via codon usage patterns. *PNAS* **118**, e2016810118, doi:10.1073/pnas.2016810118/-/DCSupplemental.y (2021).

4 Hurwitz, B. L. & Sullivan, M. B. The Pacific Ocean virome (POV): a marine viral metagenomic dataset and associated protein clusters for quantitative viral ecology. *PLoS One* **8**, e57355, doi:10.1371/journal.pone.0057355 (2013).

5 Sullivan, M. J., Petty, N. K. & Beatson, S. A. Easyfig: a genome comparison visualizer. *Bioinformatics* **27**, 1009-1010, doi:10.1093/bioinformatics/btr039 (2011).

6 Robinson, M. D., McCarthy, D. J. & Smyth, G. K. edgeR: a Bioconductor package for differential expression analysis of digital gene expression data. *Bioinformatics* **26**, 139-140, doi:10.1093/bioinformatics/btp616 (2010).

7 Xiao, X., Wang, P., Zeng, X., Bartlett, D. H. & Wang, F. *Shewanella psychrophila* sp. nov. and *Shewanella piezotolerans* sp. nov., isolated from west Pacific deep-sea sediment. *Int. J. Syst. Evol. Microbiol.* **57**, 60-65, doi:10.1099/ijs.0.64500-0 (2007).

8 Xu, G., Jian, H., Xiao, X. & Wang, F. Complete genome sequence of *Shewanella psychrophila* WP2, a deep-sea bacterium isolated from west Pacific sediment. *Marine genomics* **35**, 19-21, doi:10.1016/j.margen.2017.03.004 (2017).

9 Edwards, R. A., Keller, L. H. & Schifferli, D. M. Improved allelic exchange vectors and their use to analyze 987P fimbria gene expression. *Gene* **207**, 149-157 (1998).
