# Supplementary material for: A systematic analysis of marine lysogens and proviruses
Source: Nat Commun. 2023 Sep 27;14:6013. doi: 10.1038/s41467-023-41699-4 (PMC10533544; doi:10.1038/s41467-023-41699-4)
Supplement: Supplementary file 3 — Description of Additional Supplementary Files [file 41467_2023_41699_MOESM3_ESM.pdf]

## Description of Additional Supplementary Files:

**Supplementary Dataset 1:** Detailed information on the Marine Prokaryotic Genomes Dataset (MPGD).

**Supplementary Dataset 2:** List of temperate viruses detected by Virsorter2.

**Supplementary Dataset 3:** Quality assessment of marine temperate viruses by CheckV.

**Supplementary Dataset 4:** Detailed information on the Marine Temperate Viruses Genome Dataset (MTVGD).

**Supplementary Dataset 5:** The viral clusters grouped by vConTACT2.

**Supplementary Dataset 6:** Detailed information on the marine temperate viral clusters (tVCs) and temperate viral genera (tVG).

**Supplementary Dataset 7:** The lysogeny ratio (LyR) among marine prokaryotes at different taxonomic levels.

**Supplementary Dataset 8:** The lysogeny ratio (LyR) among marine prokaryotes in different ocean zones.

**Supplementary Dataset 9:** Descriptive statistics of the genomic features of marine lysogenic and nonlysogenic prokaryotes.

**Supplementary Dataset 10:** Predicted minimum doubling time (MDT) of marine prokaryotes.

**Supplementary Dataset 11:** Correlation analysis between the lysogeny ratio and genomic features of marine prokaryotes.

**Supplementary Dataset 12:** Comparison of the genomic features between marine temperate viruses in different ocean zones.

**Supplementary Dataset 13:** General properties of the marine temperate virus–host interaction networks.

**Supplementary Dataset 14:** General properties of the marine temperate virus–host coinfection networks.

**Supplementary Dataset 15:** Detailed information on the AMGs encoded by marine temperate viruses.

**Supplementary Dataset 16:** Enzymatic activity assays of two AMGs-encoded glycoside hydrolases.

**Supplementary Dataset 17:** Detailed information on the prediction of attachment sites.

**Supplementary Dataset 18:** tRNAs encoded by marine temperate viral genomes.

**Supplementary Dataset 19:** Comparison of the oligonucleotide frequency dissimilarity ($d_2$*) and codon cosine distance between marine temperate virus–host pairs.

**Supplementary Dataset 20:** Differentially expressed genes in WP2ΔSP1 by compared with WP2.
