# Supplementary material for: A systematic analysis of marine lysogens and proviruses
Source: Nat Commun. 2023 Sep 27;14:6013. doi: 10.1038/s41467-023-41699-4 (PMC10533544; doi:10.1038/s41467-023-41699-4)
Supplement: Supplementary file 25 — Reporting Summary [file 41467_2023_41699_MOESM25_ESM.pdf]

nature portfolio

Corresponding author(s): Huahua Jian

Last updated by author(s): Sep 6, 2023

Reporting Summary

Nature Portfolio wishes to improve the reproducibility of the work that we publish. This form provides structure for consistency and transparency in reporting. For further information on Nature Portfolio policies, see our Editorial Policies and the Editorial Policy Checklist.

Statistics

For all statistical analyses, confirm that the following items are present in the figure legend, table legend, main text, or Methods section.

n/a	Confirmed	
☐	☒	The exact sample size (*n*) for each experimental group/condition, given as a discrete number and unit of measurement
☐	☒	A statement on whether measurements were taken from distinct samples or whether the same sample was measured repeatedly
☐	☒	The statistical test(s) used AND whether they are one- or two-sided<br>*Only common tests should be described solely by name; describe more complex techniques in the Methods section.*
☒	☐	A description of all covariates tested
☐	☒	A description of any assumptions or corrections, such as tests of normality and adjustment for multiple comparisons
☐	☒	A full description of the statistical parameters including central tendency (e.g. means) or other basic estimates (e.g. regression coefficient) AND variation (e.g. standard deviation) or associated estimates of uncertainty (e.g. confidence intervals)
☐	☒	For null hypothesis testing, the test statistic (e.g. *F*, *t*, *r*) with confidence intervals, effect sizes, degrees of freedom and *P* value noted<br>*Give P values as exact values whenever suitable.*
☒	☐	For Bayesian analysis, information on the choice of priors and Markov chain Monte Carlo settings
☒	☐	For hierarchical and complex designs, identification of the appropriate level for tests and full reporting of outcomes
☐	☒	Estimates of effect sizes (e.g. Cohen's *d*, Pearson's *r*), indicating how they were calculated

Our web collection on statistics for biologists contains articles on many of the points above.

Software and code

Policy information about availability of computer code

Data collection	No software was used to collect data.
Data analysis	CheckM v1.1.3; pyani v0.2.7; GTDB-Tk v1.3.0; FastTree2 v2.1.10; iTOL v6; VirSorter2 v2.2.2; CheckV v0.8.1; DRAM-v v1.2.4; Prodigal v2.6.3; CD-HIT v4.8.1; vConTACT2 v2.0; Diamond v0.9.14.115; Cytoscape v3.7.0; R 4.0.2; R package seqinr; get_gc_and_narsc.py; R package gRodon v1.0; VirHostMatcher v1.0; tRNAscan-SE v2.0.3; Scipy v1.5.0; fastp v0.19.7; HISAT v2.1.0; RSEM v1.3.1; DEGseq v1.36.0; edgeR v3.20.2; AlphaFold v2.1; DALI online version; PyMOL v2.0; clinker v0.0.27; VIRIDIC online version; R package maps.

For manuscripts utilizing custom algorithms or software that are central to the research but not yet described in published literature, software must be made available to editors and reviewers. We strongly encourage code deposition in a community repository (e.g. GitHub). See the Nature Portfolio guidelines for submitting code & software for further information.

Data

Policy information about availability of data

All manuscripts must include a data availability statement. This statement should provide the following information, where applicable:

- Accession codes, unique identifiers, or web links for publicly available datasets
- A description of any restrictions on data availability
- For clinical datasets or third party data, please ensure that the statement adheres to our policy

All assembled prokaryotic genomes (n=12,080) used in this study were collected from publicly available databases including the NCBI GenBank (v244) as well as the Genomes from Earth’s Microbiomes (GEM) and Oceanic Trench Microbial Genomes (OTMG) datasets. Detail information of marine prokaryotic genomes and

temperate viral genomes are described and available publicly in Supplementary Data 1 and 4. The nucleotide sequences of marine temperate viral genomes (n=12,918) were deposited in the Supplementary Data 21. The transcriptomic data from the current study was deposited in the figshare (10.6084/m9.figshare.24086055). Source data are provided with this paper

Human research participants

Policy information about studies involving human research participants and Sex and Gender in Research.

Reporting on sex and gender	n/a
Population characteristics	n/a
Recruitment	n/a
Ethics oversight	n/a

Note that full information on the approval of the study protocol must also be provided in the manuscript.

Field-specific reporting

Please select the one below that is the best fit for your research. If you are not sure, read the appropriate sections before making your selection.

[x] Life sciences [ ] Behavioural & social sciences [ ] Ecological, evolutionary & environmental sciences

For a reference copy of the document with all sections, see nature.com/documents/nr-reporting-summary-flat.pdf

Life sciences study design

All studies must disclose on these points even when the disclosure is negative.

Sample size	The Marine Prokaryotic Genome dataset (MPGD) was comprised of 12,080 genomes derived from marine and with high-quality (completeness > 80% and contamination < 5%) . The Marine Temperate Viral Genome dataset (MTVGD) was comprised of 12,918 viral genomes predicted from the MPGD.
Data exclusions	Genomes from host-associated biosamples and biosamples collected at the border between marine and terrestrial environments, including the coast, beach, seashore, estuary and intertidal zone, were excluded. Viral sequences predicted from the MPGD were carefully screened and manual curated according to the recommended criteria published by Sullivan lab. 25 viral sequences > 200 kb with VirSorter2 confident score < 0.9 and 15 viral sequences annotated as ϕX174 were excluded. All lysogeny ratios (LyRs) at the class and genus levels were calculated only using classes and genera with ≥ 20 and ≥ 5 genomes, respectively. Only temperate viruses with a single-copy marker protein and their hosts were considered for analysis of polylysogeny and coinfections. Correlation analysis between prokaryotic LyRs (at the genus level) and genomic features were calculated only using genera with ≥ 5 genomes and data groups with ≥ 5 genera. The minimal doubling time (MDT) comparison between marine lysogens and nonlysogens only used the genomes with predicted MTD < 5 h.
Replication	Normally, values represent the mean ± SD of triplicate assays in this study, and all attempts at replication were successful.
Randomization	All genomes from marine biosamples were explicitly collected and predicted for temperate viral sequences, and were not relevant with randomization. Saturation analysis of protein clusters (PCs) of marine temperate viruses was based on random sampling.
Blinding	Blinding was not applicable to this study. Knowledge of marine environment was essential for analysis. The blinding was not relevant, and the exclusion of it had no effect on the accuracy and reliability of the results.

Reporting for specific materials, systems and methods

We require information from authors about some types of materials, experimental systems and methods used in many studies. Here, indicate whether each material, system or method listed is relevant to your study. If you are not sure if a list item applies to your research, read the appropriate section before selecting a response.

Materials & experimental systems

n/a	Involved in the study
☒	☐ Antibodies
☒	☐ Eukaryotic cell lines
☒	☐ Palaeontology and archaeology
☒	☐ Animals and other organisms
☒	☐ Clinical data
☒	☐ Dual use research of concern

Methods

n/a	Involved in the study
☒	☐ ChIP-seq
☒	☐ Flow cytometry
☒	☐ MRI-based neuroimaging
